# Disease burden of untreated thymidine kinase 2 deficiency: insights from a large patient dataset

**DOI:** 10.1093/braincomms/fcag200

**Published:** 2026-06-03

**Authors:** Cristina Domínguez-González, Caterina Garone, Andrés Nascimento, Yuanjun Ma, Nada Boudiaf, Richard Kim, Susan VanMeter, Marcus Brunnert, Michio Hirano

**Affiliations:** Neuromuscular Unit, Neurology Department, Hospital 12 de Octubre, Madrid 28041, Spain; Mitochondrial and Neuromuscular Research Group '12 de Octubre', imas12 Research Institute, Madrid 28041, Spain; Biomedical Network Research Centre on Rare Diseases (CIBERER), Instituto de Salud Carlos III, Madrid 28029, Spain; Department of Medical and Surgical Sciences, Alma Mater Studiorum, University of Bologna, Bologna 40138, Italy; IRCCS Istituto delle Scienze Neurologiche, UOC Neuropsichiatria dell'età Pediatrica di Bologna, Bologna 40139, Italy; Biomedical Network Research Centre on Rare Diseases (CIBERER), Instituto de Salud Carlos III, Madrid 28029, Spain; Neuromuscular Unit, Sant Joan de Déu Hospital, Barcelona 08950, Spain; Real World Evidence, UCB, Slough SL1 3WE, UK; Real World Evidence, UCB, Slough SL1 3WE, UK; Global Clinical Development, UCB, Morrisville, NC 27560, USA; Global Clinical Development, UCB, Morrisville, NC 27560, USA; Biometrics and Data Science, UCB, Monheim am Rhein 40789, Germany; Department of Neurology, H. Houston Merritt Center for Neuromuscular Disorders, Columbia University Irving Medical Center, New York, NY 10032, USA

**Keywords:** thymidine kinase 2 deficiency, mitochondrial myopathy, natural history, survival, motor milestones

## Abstract

Thymidine kinase 2 deficiency (MIM 609560) is an ultra-rare, autosomal recessive mitochondrial disease, resulting in progressive myopathy, respiratory insufficiency and increased risk of early death. Doxecitine and doxribtimine represents the first approved treatment for thymidine kinase 2 deficiency in the USA and the EU; previously, management was restricted to supportive care. The overall understanding of the natural history of thymidine kinase 2 deficiency is limited. Our study describes the baseline characteristics, survival and disease progression of untreated patients with thymidine kinase 2 deficiency as part of one of the largest international datasets to date. Data from individuals with thymidine kinase 2 deficiency identified through the review of published literature and a retrospective chart review study (NCT05017818) were pooled with pretreatment data from patients later treated with pyrimidine nucleos(t)ides (NCT03701568; NCT03845712; NCT05017818; company-supported Expanded Access Programs). Subgroups were stratified by age of thymidine kinase 2 deficiency symptom onset (≤12 years and >12 years). Key outcomes measured included survival, developmental motor milestone attainment, loss, regain and use of ventilatory and feeding support. In total, 257 patients were included in the study. Most patients [*n* = 199 (77.4%)] had an age of symptom onset ≤12 years, while 49 (19.1%) had an age of symptom onset >12 years; age of onset was missing for 9 (3.5%). Kaplan–Meier survival analyses estimated that the median time (95% confidence interval) from symptom onset to death was 2.6 (1.3, 6.4) years with age of symptom onset ≤12 years and 24.0 (16.0, not applicable) years with age of symptom onset >12 years. Loss of previously acquired motor milestones was observed across both subgroups, though most frequently in those with age of symptom onset ≤12 years [61/75 patients (81.3%) lost ≥1 motor milestone]. Spontaneous regain of lost motor milestones was rare [3/71 patients (4.2%), all with age of symptom onset ≤12 years]. Use of ventilatory support was observed for both subgroups [81/199 patients (40.7%) with age of symptom onset ≤12 years (missing data, *n* = 73); 23/49 patients (46.9%) with age of symptom onset >12 years (missing data, *n* = 11)]. Use of feeding tube support was also reported [28/199 patients (14.1%) with age of symptom onset ≤12 years (missing data, *n* = 121); 4/49 patients (8.2%) with age of symptom onset >12 years (missing data, *n* = 21)]. This study confirms the severe disease burden and high mortality associated with thymidine kinase 2 deficiency, underscoring the devastating impact on quality of life. This comprehensive dataset provides a valuable resource for informing clinical management and future therapeutic strategies.

## Teaser

Domínguez-González *et al*. studied 257 patients with thymidine kinase 2 deficiency to understand how symptoms change over time. They highlight the serious effects on daily life and life expectancy, especially for those who develop symptoms at an early age, underlining the urgent need for effective treatments for this ultra-rare disease.

## Graphical Abstract

**Graphical Abstract:**
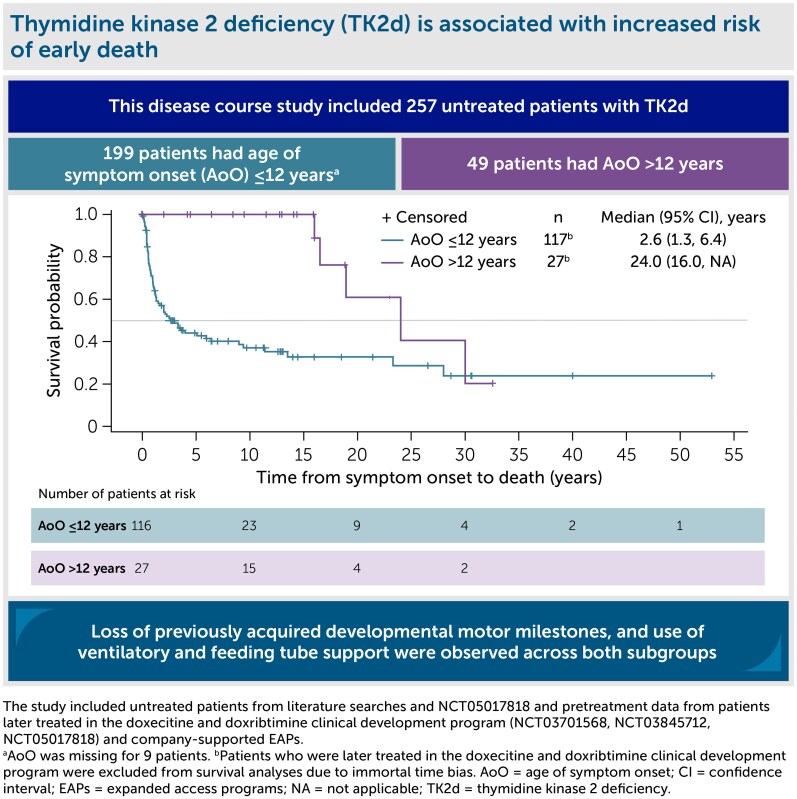

## Introduction

Thymidine kinase 2 deficiency (TK2d) (MIM 609560) is an ultra-rare, progressive, debilitating, and life-threatening genetic mitochondrial myopathy resulting from autosomal recessive pathogenic variants of the thymidine kinase 2 (*TK2*) gene.^1-6^ Such *TK2* variants in nuclear DNA lead to impaired activity of the mitochondrial matrix enzyme TK2, which phosphorylates deoxythymidine (dT) and deoxycytidine (dC) to their respective deoxynucleoside monophosphates (dNMPs). These dNMPs are further phosphorylated to generate deoxynucleoside triphosphates (dNTPs) that are incorporated into replicating mitochondrial DNA (mtDNA).^2^ The imbalance of the dNTP pool arising from impaired TK2 activity leads to mtDNA depletion and/or multiple deletions,^2,6,7^ with resulting deficiencies in mtDNA-encoded subunits of mitochondrial respiratory chain complexes that are required for cellular energy production.^3,7^ Cells with the highest energy demands are often most affected in mitochondrial diseases,^8^ and in TK2d there is a prevalent tissue-specificity for muscle, which impacts the initial presentation of symptoms.^9^

Patients with TK2d predominantly experience progressive myopathy with bulbar weakness and respiratory insufficiency, and many lose the ability to walk, eat and breathe independently.^1,10^ Although the isolated myopathic form is the most common clinical presentation, there are reports of TK2d also affecting other organs, including the central nervous system (seizures, encephalopathy, cognitive impairment), peripheral nervous system (peripheral neuropathy), heart (cardiomyopathy, arrhythmias), liver (hepatic dysfunction/failure) and kidneys (nephropathy). Involvement of other organs is most commonly reported in patients with age of TK2d symptom onset ≤12 years, with up to a third of patients with earliest onset affected. Peripheral neuropathy is more common in patients with age of symptom onset >12 years.^1,2,11^ TK2d increases the risk of early death, primarily due to respiratory failure caused by respiratory muscle weakness.^1,6^

Although it is challenging to estimate the prevalence of TK2d, current estimates suggest a prevalence of 1.64 per million people worldwide [first and third quartiles (Q1, Q3): 0.5, 3.1 per million].^5^ TK2d manifests as a continuous clinical spectrum with a varying age of symptom onset; however, it has been suggested that stratifying by age of symptom onset may be useful when describing clinical forms of the disease.^1,2,6^ Patients with age of symptom onset ≤2 years tend to experience the most rapid progression to early death and often fail to achieve expected developmental motor milestones or lose previously acquired ones. Patients with age of symptom onset >2 to ≤12 years generally experience slower progression to early death than those with age of symptom onset ≤2 years. In most cases, these patients present with loss of previously acquired motor milestones, including the ability to stand and walk within a decade, and progress to using ventilatory support. Those with age of symptom onset >12 years tend to experience the slowest progression compared with the other age-of-symptom-onset subgroups. However, variability between patients is high and natural history data in this group of patients are limited, making disease trajectory and prognosis unclear. A key feature in this patient subgroup is respiratory involvement, with most requiring ventilatory support while retaining the ability to walk.^1,2,4,12^ The age cut-off used in literature to define earlier onset forms of TK2d can differ; however, using a cut-off of ≤12 years versus >12 years for the age of symptom onset has previously been suggested as a clinically meaningful approach to disease categorization.^1,2,13^

As of November 2025 and March 2026, doxecitine and doxribtimine, an oral pyrimidine nucleoside therapy containing dC and dT, is the first approved treatment for paediatric and adult patients with TK2d with age of symptom onset ≤12 years by the US Food and Drug Administration (FDA) and the European Medicines Agency (EMA), respectively.^14-17^ Prior to this approval, management was limited to supportive care only.^18,19^

A general understanding of the natural disease course of TK2d is limited by its rarity and a lack of disease awareness, with few natural history studies performed. Prospective data are limited to a single study of adults with the disease,^20^ with no specific disease registries. Although registries are available for mitochondrial diseases, few patients with TK2d are enrolled and information may remain confined within individual registries with differing structures. As such, large datasets specific to TK2d can be hard to obtain. The objective of this study is to describe the baseline characteristics, survival and disease course (motor milestone patterns and use of ventilatory and feeding support) of patients with TK2d as part of a comprehensive, large-scale dataset.

## Materials and methods

### Study design

Various data sources were utilized to generate a global dataset of untreated patients with TK2d. A comprehensive literature search (Supplementary Table 1) for case series and case reports was conducted in June 2019 and updated in October 2021, forming the updated-Untreated Patient Database (UPD). Data were also sourced from MT-1621-107 (NCT05017818), a multicentre non-interventional chart review of untreated patients with TK2d and patients who later received pyrimidine nucleos(t)ide therapy outside of the company-sponsored clinical development programme. The dataset also included pretreatment patient data sourced from MT-1621-107, alongside MT-1621-101 (NCT03701568), TK0102 (NCT03845712), and company-supported Expanded Access Programs (EAPs). MT-1621-101 and TK0102 were phase 2 studies of patients with TK2d who received pyrimidine nucleos(t)ide therapy as part of the company-sponsored clinical development programme.^14,17^ The ongoing company-supported EAPs enable patients at risk of death or major disability to receive doxecitine and doxribtimine. Data were either collected prospectively (some patients from TK0102; company-supported EAPs) or retrospectively (all other sources). Data were cross-checked across all sources to remove duplicates to the extent possible, with the aim of including only unique patient data in the study sample (Supplementary Table 2).

Genetic confirmation of a TK2d diagnosis was required for inclusion across all data sources. An additional inclusion criterion for the updated-UPD was the availability of patient-level data. Additional inclusion and exclusion criteria have been described fully elsewhere for the individual studies in the clinical development programme^14,17^; of note, the presence of another genetic disease or polygenic disease was an exclusion criterion. Additional inclusion criteria for MT-1621-107 were the availability of medical records or, at a minimum, information pertaining to vital status, with no exclusion criteria. Company-supported EAPs comprise patients who were not eligible to participate in clinical studies. No additional exclusion criteria were applied for this study.

As de-identified data for the updated-UPD were sourced from the public domain, it was not possible to seek additional informed consent for these patients. Written informed consent was obtained for all other patients. Approvals from respective institutional review boards and ethics committees were obtained for studies in the clinical development programme, which were conducted in accordance with the Declaration of Helsinki.

### Patient population

**Figure 1:**
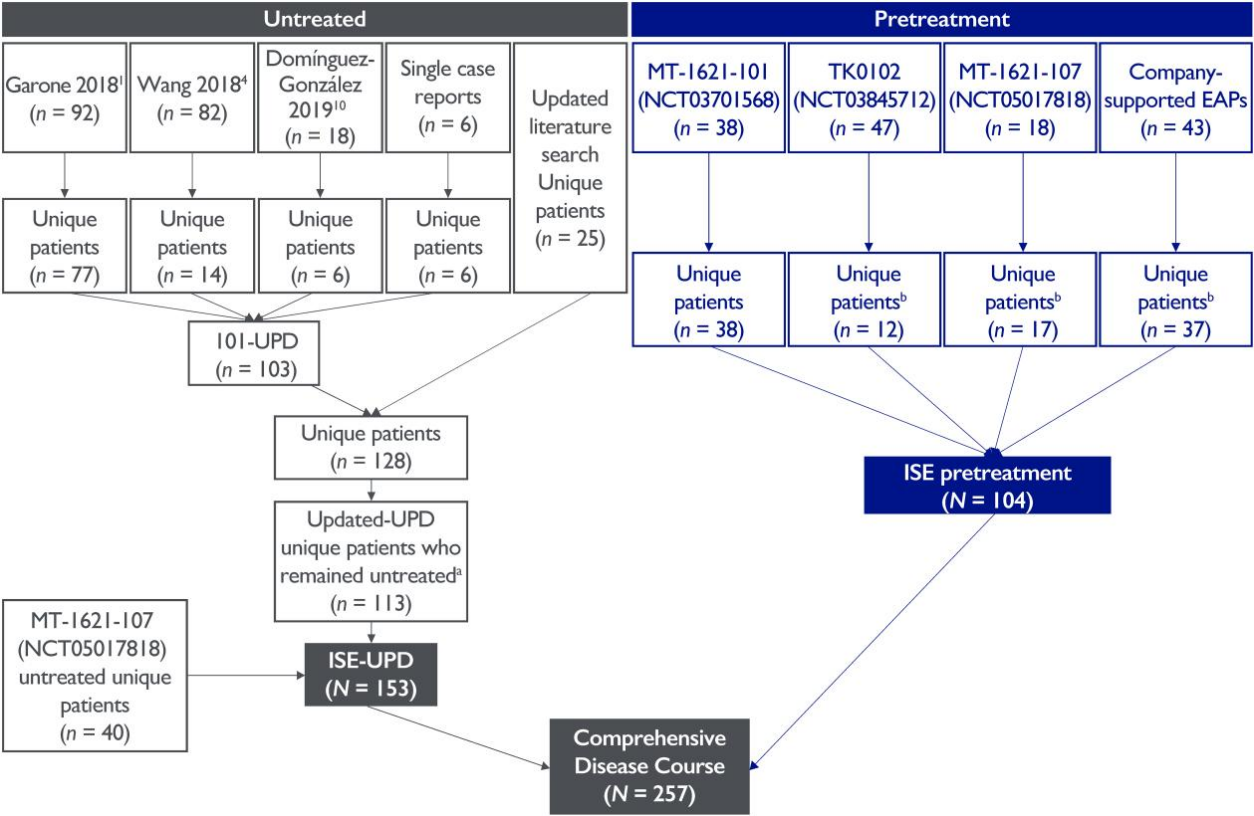
**Study analysis populations.**  ^a^Of 128 unique patients sources from literature, 1 patient without genetic confirmation of a TK2d diagnosis was excluded from the updated-UPD group, while 14 patients were excluded as they later received treatment. ^b^Patients who received treatment in another study were not included to avoid the duplication of data to the extent possible. EAP, Expanded Access Program; ISE, Integrated Summary of Efficacy; TK2d, thymidine kinase 2 deficiency; UPD, Untreated Patient Database.

The updated-UPD was pooled with untreated patients from MT-1621-107 to make up the Integrated Summary of Efficacy (ISE)-UPD. The ISE pretreatment group was formed of pretreatment data from patients who later received pyrimidine nucleos(t)ides in MT-1621-101, TK0102, MT-1621-107 or the company-supported EAPs. Finally, the ISE-UPD was combined with the ISE pretreatment group to form the Comprehensive Disease Course group (Fig. 1).

Not all outcomes were captured in all sources, and the number of patients available for analyses differed accordingly. The ISE pretreatment group was not included in the survival analyses due to immortal time bias, as patients who later received treatment, by definition, had to have remained alive over the entire study pretreatment period.

Outcomes were analysed by subgroups defined by age of symptom onset: ≤12 years and >12 years. Additionally, the ≤12 years subgroup was further analysed according to age of symptom onset ≤2 years and >2 to ≤12 years. Within age-of-symptom-onset subgroups, data from the ISE-UPD and ISE pretreatment group were also analysed separately to assess comparability of the two patient populations.

### Outcomes

Owing to the rare nature of this disease and the limited availability of information on TK2d, this study includes information collected through both cross-sectional and longitudinal methods. Objective clinical and functional outcomes with defined discrete variables and clear-cut events relevant to TK2d were analysed to mitigate potential variability arising from methodological differences. Relevant outcome measures identified for data collection included survival, and the attainment, loss and regain of key developmental motor milestones reflective of those described by the World Health Organization.^21^ The developmental motor milestones examined were the following: hold head upright; sit upright; stand, assisted and unassisted; walk, assisted and unassisted; climb stairs, assisted and unassisted and run. The proportion of patients using ventilatory support was examined, as well as age at first use, mode [invasive, with tracheostomy or no tracheostomy, or non-invasive, with bilevel positive airway pressure (BiPAP) or continuous positive airway pressure (CPAP)] and amount of support used. In addition, the proportion of patients using enteral feeding support (nasogastric/gastrostomy tube) at any time or long term was examined, as well as age at first use and reason for tube insertion.

### Statistical analyses

Data for demographics, clinical characteristics, and survival were assessed using descriptive statistics. Continuous variables were summarized using mean, standard deviation, median, minimum, maximum, and first and third quartiles (Q1 and Q3), while categorical variables were summarized using frequency and proportions.

The Kaplan–Meier method was applied to analyse time-to-event outcomes for the time from birth and TK2d symptom onset to death, first motor milestone loss, first use of ventilatory and first use of feeding support. Median time-to-event along with respective first and third quartiles (Q1 and Q3) and 95% confidence intervals (CIs) were estimated. Patients for whom event data were not collected nor had missing dates were censored at time point zero. For time to death, patients who were still alive at last follow-up were censored at their age last known alive. In all other time-to-event analyses, patients who did not experience the event were censored at age last known alive or first treated (if applicable), whichever occurred first.

Imputation of missing or partial dates followed rules set forth in the statistical analysis plans of the original studies; no other data imputation was performed, and analyses were based on observed cases only. All statistical analyses were performed using SAS software, version 9.4 (SAS institute Inc., Cary, NC, USA).

## Results

### Patient baseline demographics and characteristics

Overall, 257 patients were included in the Comprehensive Disease Course group, with 153 untreated patients from the ISE-UPD, and 104 patients from the ISE pretreatment group. Of the 257 patients, the majority [*n* = 199 (77.4%)] had an age of symptom onset ≤12 years, and 49 (19.1%) had an age of symptom onset >12 years. The remaining 9 patients (3.5%) did not have data on symptom onset. Due to the retrospective data collection methods used for parts of the study population, there was a proportion of missing data throughout, particularly in the updated-UPD for which no race and ethnicity data were available. Nonetheless, baseline demographics and characteristics of the ISE-UPD and ISE pretreatment group were broadly comparable for patients with age of symptom onset ≤12 years and >12 years (Table 1; MT-1621-107 untreated and updated-UPD groups shown in Supplementary Table 3). An exception was that, for patients with age of symptom onset >12 years, those in the ISE-UPD had a median age of symptom onset over 10 years older than those in the ISE pretreatment group. Of those with age of symptom onset ≤12 years, the majority [*n* = 146 (73.4%)] were aged ≤2 years at symptom onset. Baseline demographics and characteristics remained largely comparable for the ISE-UPD and ISE pretreatment group across the age-of-symptom-onset-≤2 years and >2 to ≤12 years subgroups (Supplementary Table 3, including MT-1621-107 untreated and updated-UPD groups).

**Table 1:** Baseline demographics and characteristics for patients with age of TK2d symptom onset ≤12 years and >12 years

	Patients with age of TK2d symptom onset ≤12 years^a^			Patients with age of TK2d symptom onset >12 years^a^		
	ISE-UPD	ISE pretreatment	Comprehensive Disease Course	ISE-UPD	ISE pretreatment	Comprehensive Disease Course
***N***	*117*	*82*	*199*	*27*	*22*	*49*
**Sex, *n* (%)**						
Male	62 (53.0)	46 (56.1)	108 (54.3)	9 (33.3)	9 (40.9)	18 (36.7)
Female	53 (45.3)	36 (43.9)	89 (44.7)	16 (59.3)	13 (59.1)	29 (59.2)
Missing	2 (1.7)	0 (0)	2 (1.0)	2 (7.4)	0 (0)	2 (4.1)
**Race,^b^ *n* (%)**						
White	24 (20.5)	67 (81.7)	91 (45.7)	10 (37.0)	20 (90.9)	30 (61.2)
Other	2 (1.7)	11 (13.4)	13 (6.5)	0 (0)	2 (9.1)	2 (4.1)
Missing	91 (77.8)	4 (4.9)	95 (47.7)	17 (63.0)	0 (0)	17 (34.7)
**Ethnicity, *n* (%)**						
Hispanic or Latino	12(10.3)	30 (36.6)	42 (21.1)	1 (3.7)	0 (0)	1 (2.0)
Not Hispanic or Latino	14 (12.0)	41 (50.0)	55 (27.6)	10 (37.0)	20 (90.9)	30 (61.2)
Missing	91 (77.8)	11 (13.4)	102 (51.3)	16 (59.3)	2 (9.1)	18 (36.7)
**Geographic region of residence,^b^ *n* (%)**						
Europe	20 7.1)	27 (32.9)	47 (23.6)	12 (44.4)	16 (72.7)	28 (57.1)
Rest of world	48 (41.0)	55 (67.1)	103 (51.8)	4 (14.8)	6 (27.3)	10 (20.4)
Missing	49 (41.9)	0 (0)	49 (24.6)	11 (40.7)	0 (0)	11 (22.4)
**Age of TK2d symptom onset, years**	*n* = 117	*n* = 82	*n* = 199	*n* = 27	*n* = 22	*n* = 49
Median (min, max)	1.2 (0.0, 11.0)	1.5 (0.0, 11.7)	1.4 (0.0, 11.7)	40.0 (12.0, 72.0)	27.1 (12.4, 60.3)	31.0 (12.0, 72.0)
Q1, Q3	0.5, 2.0	1.1, 2.4	0.8, 2.3	23.5, 45.0	17.8, 40.0	20.0, 40.0
**Age at genetic confirmation, years**	*n* = 59	*n* = 77	*n* = 136	*n* = 16	*n* = 22	*n* = 38
Median (min, max)	5.2 (0.0, 56.4)	3.2 (0.1, 35.3)	4.1 (0.0, 56.4)	44.9 (22.0, 75.5)	48.1 (15.0, 73.6)	46.3 (15.0, 75.5)
Q1, Q3	2.0, 14.4	1.6, 8.3	1.7, 10.3	39.9, 53.7	29.6, 57.8	30.4, 56.8
**Time from TK2d symptom onset to genetic confirmation, months**	*n* = 59	*n* = 77	*n* = 136	*n* = 16	*n* = 22	*n* = 38
Median (min, max)	38.1 (−5.9, 556.4)	12.3 (−59.9, 359.9)	24.7 (−59.9, 556.4)	152.8 (17.4, 358.3)	172.5 (3.5, 524.0)	159.1 (3.5, 524.0)
Q1, Q3	9.4, 129.1	4.3, 64.7	6.3, 90.1	80.6, 216.2	119.0, 311.1	97.0, 271.4

^a^Age of TK2d symptom onset could not be determined for nine patients; therefore, their data could not be included in this table.

^b^Owing to the ultra-rare nature of TK2d and the small number of patients, some details relating to race and country of residence were grouped for reporting purposes to minimize risk of patient identification. ISE, Integrated Summary of Efficacy; max, maximum; min, minimum; Q1, quartile 1; Q3, quartile 3; TK2d, thymidine kinase 2 deficiency; UPD, Untreated Patient Database.

Of patients in the Comprehensive Disease Course subgroup with age of symptom onset ≤12 years, 54.3% were males and 44.7% were females, and 51.8% of patients resided outside of Europe (Table 1). For patients in the Comprehensive Disease Course group with age of symptom onset >12 years, 59.2% were female and 57.1% resided in Europe (Table 1).

Among all patients in the Comprehensive Disease Course group with available data, the median (Q1, Q3) age of symptom onset was 1.7 (0.9, 5.3) years. A delay was often observed between the onset of TK2d symptoms and genetic confirmation of the disease. The median diagnostic delay (time from symptom onset to genetic confirmation) was longest within the subgroup of patients with age of symptom onset >12 years (159.1 months) and shortest for those with age of symptom onset ≤2 years (17.5 months); the median diagnostic delay was 62.9 months for patients with age of symptom onset >2 to ≤12 years (Table 1; Supplementary Table 3).

### Survival

Survival outcomes were analysed descriptively and using Kaplan–Meier estimates from patients in the ISE-UPD (Table 2). Descriptive analyses of survival indicate that TK2d was associated with early death. Of the 117 patients in the ISE-UPD with age of symptom onset ≤12 years, 66 (56.4%) had died with a median (Q1, Q3) age at death of 1.9 (1.0, 3.5) years. Within this age-of-symptom-onset subgroup, 60/90 patients with age of symptom onset ≤2 years (66.7%) and 6/27 patients with age of symptom onset >2 to ≤12 years (22.2%) died (Supplementary Table 4). In patients with age of symptom onset >12 years in the ISE-UPD, 6/27 (22.2%) died, with a median (Q1, Q3) age at death of 64.0 (56.0, 67.0) years.

**Table 2:** Summary of survival analysis for patients in the ISE-UPD group with age of TK2d symptom onset ≤12 years and >12 years

Summary of time to death	ISE-UPD total (*n* = 153)	Age of TK2d symptom onset	
		≤12 years (*n* = 117)	>12 years (*n* = 27)
**Patient status, *n* (%)**			
Alive	65 (42.5)	41 (35.0)	19 (70.4)
Deceased	74 (48.4)	66 (56.4)	6 (22.2)
Missing data	14 (9.2)	10 (8.5)	2 (7.4)
**Age at death, years**	*n* = 73	*n* = 66	*n* = 5
Mean (SD)	7.7 (15.5)	3.8 (5.8)	59.4 (12.0)
Median (min, max)	2.0 (0.0, 70.0)	1.9 (0.0, 33.5)	64.0 (40.0, 70.0)
Q1, Q3	1.2, 4.5	1.0, 3.5	56.0, 67.0
**Kaplan–Meier estimates for time from birth to death**			
Q1 (95% CI), years	1.9 (1.5, 3.0)	1.6 (1.1, 1.9)	64.0 (40.0, 67.0)
Median (95% CI), years	13.0 (4.5, 64.0)	4.0 (2.8, 10.0)	67.0 (56.0, NA)
Q3 (95% CI), years	67.0 (56.0, NA)	33.5 (16.0, NA)	70.0 (64.0, NA)
Patients censored, n (%)	80 (52.3)	51 (43.6)	22 (81.5)
**Kaplan–Meier estimates for time from TK2d symptom onset to death**			
Q1 (95% CI), years	1.0 (0.6, 1.3)	0.7 (0.5, 1.0)	18.9 (16.0, 24.0)
Median (95% CI), years	9.0 (2.6, 18.9)	2.6 (1.3, 6.4)	24.0 (16.0, NA)
Q3 (95% CI), years	30.0 (23.3, NA)	28.0 (13.5, NA)	30.0 (18.9, NA)
Patients censored, *n* (%)	82 (53.9)^a^	51 (44.0)^a^	22 (81.5)

^a^One patient was not at risk at point in time 0.

CI, confidence interval; ISE, Integrated Summary of Efficacy; max, maximum; min, minimum; NA, not applicable; Q1, quartile 1; Q3, quartile 3; SD, standard deviation; TK2d, thymidine kinase 2 deficiency; UPD, Untreated Patient Database.

**Figure 2:**
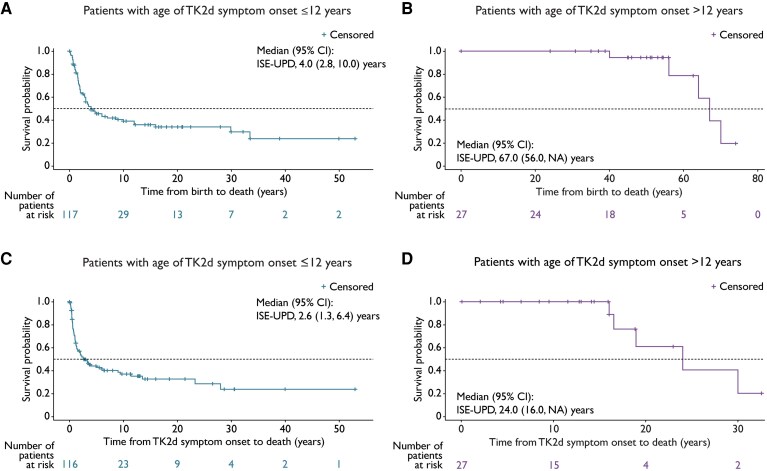
**Kaplan–Meier plots of time to death from birth and from symptom onset in patients with TK2d from the ISE-UPD.** (**A**) Estimated survival time from birth to death in patients with age of symptom onset ≤12 years in the ISE-UPD group (*n* = 117); (**B**) Estimated survival time from birth to death in patients with age of symptom onset >12 years in the ISE-UPD group (*n* = 27); (**C**) Estimated survival time from symptom onset to death in patients with age of symptom onset ≤12 years in the ISE-UPD group (*n* = 116); (**D**) Estimated survival time from symptom onset to death in patients with age of symptom onset >12 years in the ISE-UPD group (*n* = 27). CI, confidence interval; ISE, Integrated Summary of Efficacy; NA, not applicable; TK2d, thymidine kinase 2 deficiency; UPD, Untreated Patient Database.

Kaplan–Meier analyses of time from birth to death (Fig. 2A and B) and from symptom onset to death (Fig. 2C and D) in the ISE-UPD indicate that TK2d decreased the probability of survival regardless of age of symptom onset (separate curves for the MT-1621-107 untreated group and updated-UPD are shown in Supplementary Fig. 1). For patients with age of symptom onset ≤12 years, the estimated median (95% CI) time to death from birth and from symptom onset was 4.0 (2.8, 10.0) years and 2.6 (1.3, 6.4) years, respectively. For patients with age of symptom onset >12 years, the estimated median (95% CI) time to death from birth and from symptom onset was 67.0 [56.0, not applicable (NA)] years and 24.0 (16.0, NA) years, respectively (Table 2). Descriptive and Kaplan–Meier estimates of survival vary slightly owing to the differing analytical approaches.

### Developmental motor milestones

Developmental motor milestones initially achieved and subsequently lost were assessed for patients in the Comprehensive Disease Course group (Table 3). However, it should be noted that data were missing for patients in the updated-UPD. Consequently, Kaplan–Meier analyses of time to first loss of any motor milestone from birth and from symptom onset are presented for the MT-1621-107 untreated and ISE pretreatment groups.

**Table 3:** Summary of motor milestones initially achieved by patients in the Comprehensive Disease Course group^a^ with age of TK2d symptom onset ≤12 years and >12 years, and those lost over the course of disease progression

Developmental motor milestone summary	Age of TK2d symptom onset	
	≤12 years (*n* = 199)	>12 years (*n* = 49)
**Patients with data for at least one response for milestones initially achieved, *n* (%)**	78 (39.2)	28 (57.1)
Missing data	121^a^ (60.8)	21^a^ (42.9)
**Patients with number of milestones initially achieved, *n* (%)**		
≥1 milestone	75 (96.2)	28 (100)
1 milestone	5 (6.4)	1 (3.6)
2 milestones	3 (3.8)	0 (0)
3 milestones	7 (9.0)	0 (0)
≥4 milestones	60 (76.9)	27 (96.4)
**Patients with milestones initially achieved, *n*/N (%)**		
Ability to hold head upright, unassisted	72/73 (98.6)	27/27 (100)
Ability to sit upright, unassisted	67/72 (93.1)	27/27 (100)
Ability to stand, assisted	62/69 (89.9)	27/27 (100)
Ability to stand, unassisted	59/70 (84.3)	27/27 (100)
Ability to walk, assisted	56/71 (78.9)	27/27 (100)
Ability to walk, unassisted	60/75 (80.0)	28/28 (100)
Ability to climb stairs, assisted	45/69 (65.2)	27/27 (100)
Ability to climb stairs, unassisted	27/69 (39.1)	27/27 (100)
Ability to run	29/68 (42.6)	26/27 (96.3)
**Patients with data for assessing milestone loss,^b^ *n* (%)**	75 (37.7)	28 (57.1)
Missing data	124^a^ (62.3)	21^a^ (42.9)
**Patients with milestones lost, *n* (%)**		
≥1 milestone	61 (81.3)	10 (35.7)
1 milestone	12 (16.0)	7 (25.0)
2 milestones	13 (17.3)	3 (10.7)
3 milestones	8 (10.7)	0 (0)
≥4 milestones	28 (37.3)	0 (0)
**Age at which first milestone was lost, years**	*n* = 53	*n* = 7
Mean (SD)	4.3 (5.6)	41.7 (24.9)
Median (min, max)	2.0 (0.5, 27.3)	50.0 (12.5, 73.3)
Q1, Q3	1.2, 4.5	18.0, 68.0
**Milestones lost, *n* (%)**		
Ability to hold head upright, unassisted	34/72 (47.2)	0/27 (0)
Ability to sit upright, unassisted	27/67 (40.3)	0/27 (0)
Ability to stand, assisted	28/62 (45.2)	0/27 (0)
Ability to stand, unassisted	28/59 (47.5)	0/27 (0)
Ability to walk, assisted	27/56 (48.2)	0/27 (0)
Ability to walk, unassisted	31/60 (51.7)	1/28 (3.6)
Ability to climb stairs, assisted	27/45 (60.0)	0/27 (0)
Ability to climb stairs, unassisted	22/27 (81.5)	3/27 (11.1)
Ability to run	22/29 (75.9)	9/26 (34.6)

^a^Data are presented for the Comprehensive Disease Course group; however, motor milestone data were not available for any patients in the updated-UPD (age of symptom onset ≤12 years, *n* = 91; age of symptom onset >12 years, *n* = 15) and for some patients in the MT-1621-107 untreated group (age of symptom onset ≤12 years, *n* = 0; age of symptom onset >12 years, *n* = 1) and ISE pretreatment group (age of symptom onset ≤12 years, *n* = 30; age of symptom onset >12 years, *n* = 5).

^b^Patients needed to have initially achieved at least one milestone to be assessed for milestone loss.

ISE, Integrated Summary of Efficacy; max, maximum; min, minimum; Q1, quartile 1; Q3, quartile 3; SD, standard deviation; TK2d, thymidine kinase 2 deficiency; UPD, Untreated Patient Database.

**Figure 3:**
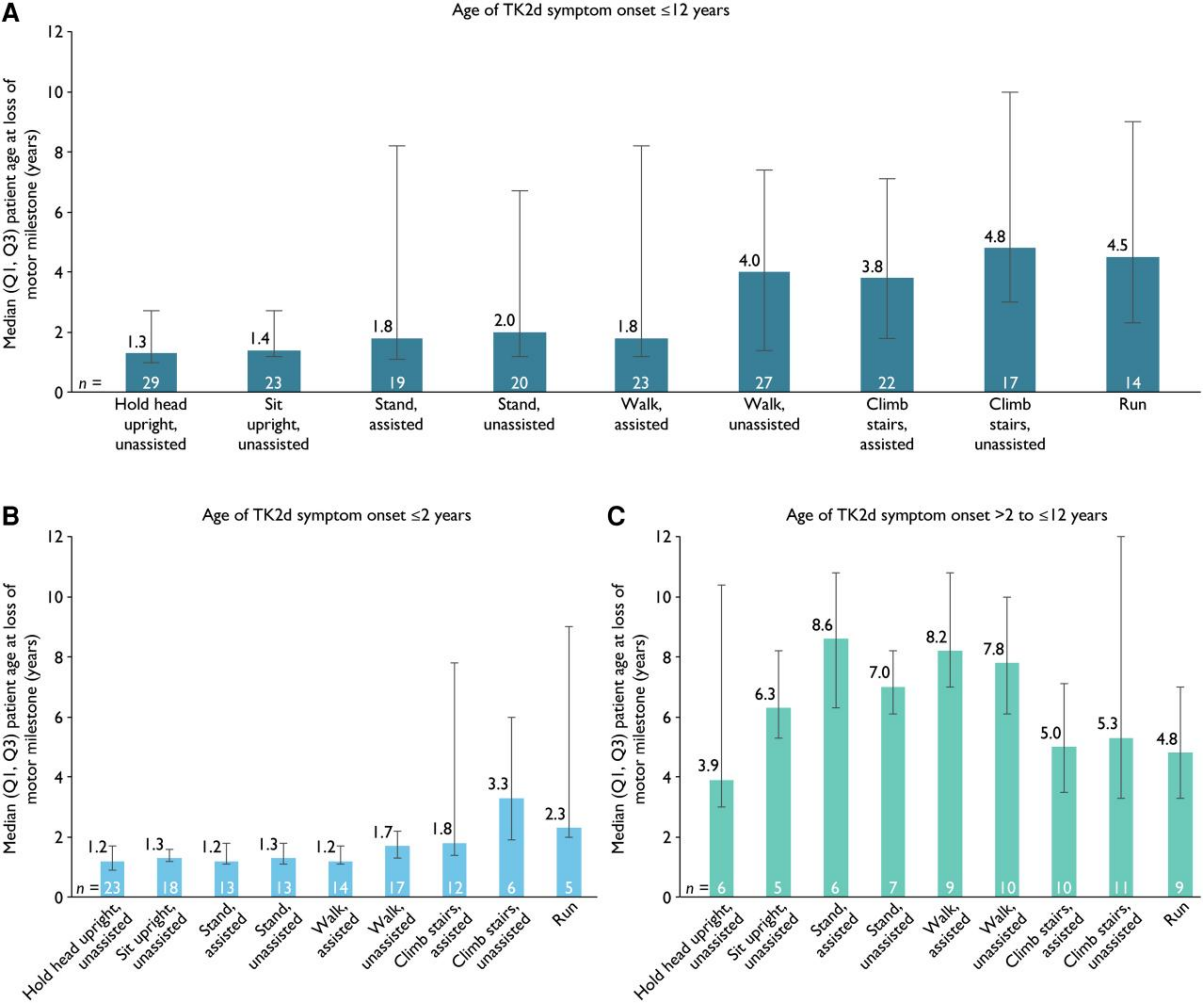
**Median age at loss of developmental motor milestones in patients in the Comprehensive Disease Course group.** (**A**) Median age at loss of each of the developmental motor milestones assessed in patients with age of symptom onset ≤12 years; (**B**) Median age at loss of each of the developmental motor milestones assessed in patients with age of symptom onset ≤2 years; (**C**) Median age at loss of each of the developmental motor milestones assessed in patients with age of symptom onset >2 to ≤12 years. Error bars show Q1 and Q3 for patient age at loss of motor milestone. Q1, quartile 1; Q3, quartile 3; TK2d, thymidine kinase 2 deficiency.

**Figure 4:**
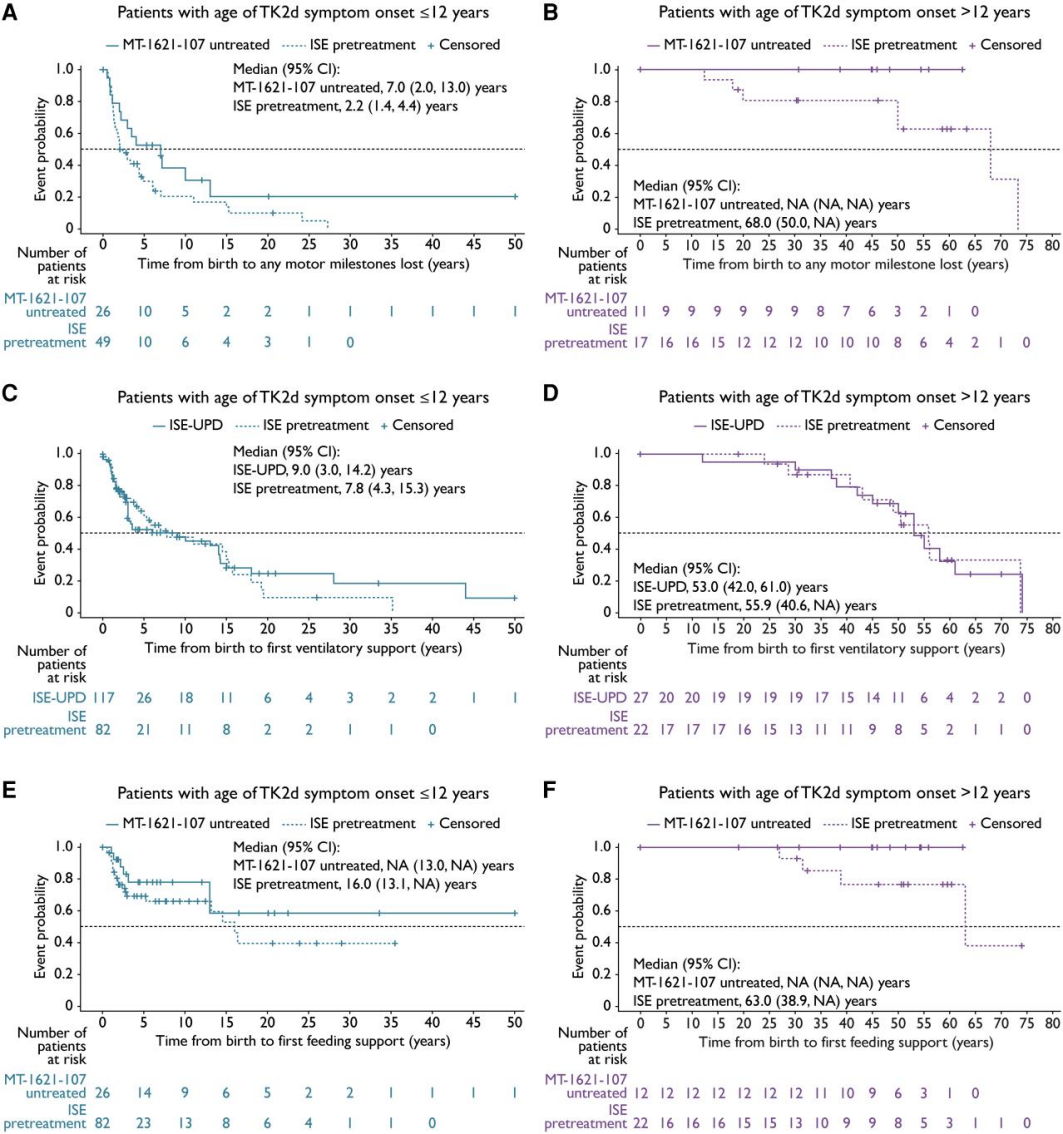
**Kaplan–Meier plots of time from birth to first loss of any motor milestone,^a^ first use of ventilatory support and first use of feeding support.** ^a^ (**A**) Estimated time from birth to any motor milestone lost in patients with age of symptom onset ≤12 years (MT-1621-107 untreated, *n* = 26; ISE pretreatment, *n* = 49); (**B**) Estimated time from birth to any motor milestone lost in patients with age of symptom onset >12 years (MT-1621-107 untreated, *n* = 11; ISE pretreatment, *n* = 17); (**C**) Estimated time from birth to first ventilatory support in patients with age of symptom onset ≤12 years (ISE-UPD, *n* = 117; ISE pretreatment, *n* = 82); (**D**) Estimated time from birth to first ventilatory support in patients with age of symptom onset >12 years (ISE-UPD, *n* = 27; ISE pretreatment, *n* = 22); (**E**) Estimated time from birth to first feeding support in patients with age of symptom onset ≤12 years (MT-1621-107 untreated, *n* = 26; ISE pretreatment, *n* = 82); (**F**) Estimated time from birth to first feeding support in patients with age of symptom onset >12 years (MT-1621-107 untreated, *n* = 12; ISE pretreatment, *n* = 22). ^a^Within the ISE-UPD, motor milestone data and feeding support data were not available for any patients in the updated-UPD; consequently, only data for the MT-1621-107 untreated group are shown for these outcomes. CI, confidence interval; ISE, Integrated Summary of Efficacy; NA, not applicable; TK2d, thymidine kinase 2 deficiency; UPD, Untreated Patient Database.

Nearly all patients with age of symptom onset ≤12 years achieved at least one of the nine assessed motor milestones [75/78 (96.2%)] and most achieved four or more [60/78 (76.9%); Table 3]. Loss of previously acquired motor milestones was prevalent in this age-of-symptom-onset subgroup, with 61/75 (81.3%) losing at least one milestone and 28/75 (37.3%) losing four or more. The most basic motor milestone evaluated, the ability to hold head upright, was lost by 34/72 patients (47.2%) with age of symptom onset ≤12 years. First milestone loss in this subgroup was typically experienced in early childhood, with a median (Q1, Q3) age at first loss of 2.0 (1.2, 4.5) years. Of patients with age of symptom onset ≤12 years who had achieved the ability to sit upright unassisted, 40% later lost that milestone at a median age of 1.4 years (Fig. 3A). Additionally, approximately half of the patients with age of symptom onset ≤12 years who achieved the abilities to stand or walk unassisted later lost these abilities. Kaplan–Meier estimates of median time (95% CI) from birth to losing a motor milestone for patients with age of symptom onset ≤12 years were 7.0 (2.0, 13.0) years in the MT-1621-107 group and 2.2 (1.4, 4.4) years in the ISE pretreatment group (Fig. 4A). Most patients started to lose milestones within the first few years of symptom onset (Supplementary Fig. 2).

For patients with age of onset ≤2 years, 32/50 (64.0%) achieved four or more motor milestones. All 28 patients (100%) with age of symptom onset from >2 to ≤12 years achieved four or more motor milestones; however, several milestones were still not universally achieved in this subgroup [most frequently, the abilities to climb stairs unassisted (7/26 patients; 26.9%) and run (6/26 patients; 23.1%); Supplementary Table 5]. Similar patterns of progressive motor milestone loss were observed for the age-of-symptom-onset ≤2 years and >2 to ≤12-years subgroups, with 38.3% and 35.7% of these patients losing four or more milestones, respectively (Supplementary Table 5). For patients with age of symptom onset ≤2 years, the loss of assessed motor milestones occurred through the median ages of 1.2–3.3 years (Fig. 3B). For those with age of symptom onset >2 to ≤12 years, loss of the abilities to hold head upright, climb stairs and run occurred through the median ages of 3.9–5.3 years; the remaining milestones were lost through the ages of 6.3–8.6 years (Fig. 3C).

Of the 28 patients with age of symptom onset >12 years and available data, 27 (96.4%) initially achieved four or more motor milestones (Table 3); data were limited for the remaining patient, though they were known to have achieved one motor milestone. Motor milestone loss was less common in this age-of-symptom-onset subgroup than in the others, though still observed. Loss of at least one milestone was seen for 10/28 (35.7%) patients, at a median (Q1, Q3) age of 50.0 (18.0, 68.0) years for first loss. The abilities to run, climb stairs unassisted or walk unassisted were lost most frequently. Kaplan–Meier estimates of time from symptom onset were limited for patients with age of symptom onset >12 years by the small number of events, particularly in the MT-1621-107 untreated group. However, for those in the ISE pretreatment population, the estimated median time (95% CI) from birth to losing a motor milestone was 68.0 (50.0, NA) years, but in some cases occurred as early as during the first 15 years of life (Fig. 4B).

Of the 71 patients in the Comprehensive Disease Course group who lost at least one motor milestone, spontaneous regain of lost motor milestones occurred in just three patients (4.2%), all with age of symptom onset ≤12 years. While overall these three patients experienced declining motor function, one regained the ability to stand assisted, one regained the ability to walk unassisted and one regained the ability to run (Supplementary Table 6).

### Ventilatory and feeding tube support

In the Comprehensive Disease Course group, ventilatory support was used in 81/199 patients (40.7%) with age of symptom onset ≤12 years and 23/49 patients (46.9%) with age of symptom onset >12 years (Table 4). In those with age of symptom onset ≤12 years, the median (Q1, Q3) age at first use was 3.0 (1.3, 10.0) years. Many of these patients used ventilatory support for a large portion of the day, with a median (Q1, Q3) usage of 12.0 (10.0, 24.0) hours per day. In these patients, non-invasive BiPAP/CPAP was the most common mode of ventilatory support (28/81 patients; 34.6%) and 15/81 patients (18.5%) used invasive support, with or without tracheostomy. A similar proportion of patients using ventilatory support was observed between the age-of-symptom-onset ≤2 years and >2 to ≤12-years subgroups (Supplementary Table 7). Similar proportions of patients with age of symptom onset ≤2 years used invasive (20.7%) and non-invasive support (29.3%). For patients with age of symptom onset >2 to ≤12 years, 13.0% used invasive and 47.8% used non-invasive support. Patients with age of symptom onset ≤2 years using ventilatory support often did so for most of the day, with a median (Q1, Q3) usage of 23.0 (10.0, 24.0) hours per day. In patients with age of symptom onset >12 years, the median (Q1, Q3) age at first use was 49.5 (38.0, 55.9) years and the median (Q1, Q3) usage per day was 8.0 (8.0, 10.0) hours. In these patients, all recorded ventilatory support used was non-invasive. Ventilatory support data were missing for 73/199 patients (36.7%) with age of symptom onset ≤12 years and 11/49 patients (22.4%) with age of symptom onset >12 years.

**Table 4:** Summary of ventilatory support and feeding support for patients in the Comprehensive Disease Course group^a^ with age of TK2d symptom onset ≤12 years and >12 years

Ventilatory and feeding tube support	Age of TK2d symptom onset	
	≤12 years (*n* = 199)	>12 years (*n* = 49)
**Ventilatory support used at any time, *n* (%)**	81 (40.7)	23 (46.9)
Missing ventilatory support data	73 (36.7)	11 (22.4)
**Age at first ventilatory support, years**	*n* = 73	*n* = 22
Mean (SD)	6.6 (8.4)	46.8 (15.0)
Median (min, max)	3.0 (0.0, 44.0)	49.5 (12.0, 74.0)
Q1, Q3	1.3, 10.0	38.0, 55.9
**Mode of first ventilatory support, *n* (%)**	*n* = 81	*n* = 23
Invasive (tracheostomy or no tracheostomy)	15 (18.5)	0 (0)
Non-invasive (e.g. BiPAP, CPAP)	28 (34.6)	16 (69.6)
Missing data	38 (46.9)	7 (30.4)
**Amount of ventilatory support used, hours/day**	*n* = 36	*n* = 14
Mean (SD)	16.3 (7.2)	9.9 (4.4)
Median (min, max)	12.0 (8.0, 24.0)	8.0 (7.0, 24.0)
Q1, Q3	10.0, 24.0	8.0, 10.0
**Feeding tube (gastrostomy/nasogastric) support used at any time, *n* (%)**	28 (14.1)	4 (8.2)
Missing feeding support data	121^a^ (60.8)	21^a^ (42.9)
**Age at first feeding support, years**	*n* = 27	*n* = 4
Mean (SD)	4.6 (5.4)	40.1 (16.0)
Median (min, max)	1.9 (0.5, 16.3)	35.2 (27.0, 63.0)
Q1, Q3	1.2, 5.2	29.2, 51.0
**Tube insertion reason for first occurrence, *n* (%)**	*n* = 28	*n* = 4
Supplemental oral intake	4 (14.3)	0 (0)
Dysphagia	13 (46.4)	4 (100)
Dysphagia, supplemental oral intake	6 (21.4)	0 (0)
Other	5 (17.9)	0 (0)
Missing data	0 (0)	0 (0)
**Long-term use of feeding tube,^b^ *n* (%)**	17 (60.7)	2 (50.0)

^a^Data are presented for the Comprehensive Disease Course group; however, feeding support data were not available for any patients in the updated-UPD (age of symptom onset ≤12 years, *n* = 91; age of symptom onset >12 years, *n* = 15) and for some patients in the ISE pretreatment group (age of symptom onset ≤12 years, *n* = 30; age of symptom onset >12 years, *n* = 6).

^b^Long-term was defined as usage for over a month.

BiPAP, bilevel positive airway pressure; CPAP, continuous positive airway pressure; max, maximum; min, minimum; Q1, quartile 1; Q3, quartile 3; SD, standard deviation; TK2d, thymidine kinase 2 deficiency.

Kaplan–Meier analyses of time to first ventilatory support from birth were performed for the ISE-UPD and ISE pretreatment groups. In the age of symptom onset ≤12 years subgroup, the estimated median (95% CI) times from birth to first use of ventilatory support in these analysis populations were 9.0 (3.0, 14.2) years and 7.8 (4.3, 15.3) years, respectively (Fig. 4C; separate curves for the MT-1621-107 untreated group and updated-UPD are shown in Supplementary Fig. 3E). The statistical risk of using ventilatory support began shortly after symptom onset and continued to accumulate over the subsequent 20 years of disease duration across analysis populations (Supplementary Fig. 3A and C). In patients with age of symptom onset >12 years, the estimated median (95% CI) times from birth to first use of ventilatory support were 53.0 (42.0, 61.0) years and 55.9 (40.6, NA) years in the ISE-UPD and ISE pretreatment groups, respectively (Fig. 4D; separate curves for the MT-1621-107 untreated group and updated-UPD are shown in Supplementary Fig. 3F). While a lower statistical risk of using ventilatory support was observed during the first 10 years after symptom onset, it continued to increase through 20 years after symptom onset (Supplementary Fig. 3B and D).

In the Comprehensive Disease Course group, enteral feeding tube support was used for 28/199 patients (14.1%) with age of symptom onset ≤12 years and 4/49 patients (8.2%) with age of symptom onset >12 years (Table 4). In those with age of symptom onset ≤12 years, median (Q1, Q3) age at first use was 1.9 (1.2, 5.2) years, most commonly to manage dysphagia. A feeding tube was used long term (defined as usage for over a month) in 17/28 patients (60.7%) in this subgroup; data on long-term use were missing for the remaining 11 patients. The proportion of patients using a feeding tube was similar for those with age of symptom onset ≤2 years [23/146 patients (15.8%)] and those with age of symptom onset >2 to ≤12 years [5/53 patients (9.4%)] (Supplementary Table 7). Dysphagia remained a common reason for tube insertion for both these age-of-symptom-onset subgroups based on available data. In those with age of symptom onset >12 years, median (Q1, Q3) age at first feeding tube use was 35.2 (29.2, 51.0) years. Dysphagia was the reason for use in all four cases in this age-of-symptom-onset subgroup and a feeding tube was used long term in 2/4 patients [50.0%; 2/4 patients (50.0%) missing data]. Data for feeding tube support were missing for 121/199 patients (60.8%) with age of symptom onset ≤12 years and for 21/49 patients (42.9%) with age of symptom onset >12 years, including for all patients in the updated-UPD (Table 4).

Owing to missing data in the ISE-UPD, Kaplan–Meier analyses of time to first use of a feeding tube from birth and from symptom onset are presented for the MT-1621-107 untreated and ISE pretreatment groups. For patients with age of symptom onset ≤12 years in the MT-1621-107 untreated and ISE pretreatment groups, the increased likelihood of using a feeding tube began in the first 5 years after both birth (Fig. 4E) and symptom onset (Supplementary Fig. 4A). For those with age of symptom onset >12 years, no patients in the MT-1621-107 untreated group had a feeding tube inserted. For those in the ISE pretreatment group, there was a delay of ∼25 years after birth (Fig. 4F) and ∼10 years after symptom onset (Supplementary Fig. 4B) before the likelihood of using a feeding tube began to increase.

The timing of initiation of ventilatory and/or feeding tube support in relation to milestone loss was assessed for patients with age of symptom onset ≤12 years. Thirty-one patients with reported use of ventilatory support, feeding support, or both, initially achieved all of the abilities to sit upright unassisted and stand and walk both assisted and unassisted. Of these patients, 38.7% (12/31) lost all these abilities before the initiation of ventilatory and/or feeding tube support (54.8% without information for loss of milestones).

## Discussion

This study, combining geographically diverse data from 257 patients, is one of the largest of its kind to date in TK2d and describes the disease burden and trajectory in affected patients. We pooled data from multiple retrospective and prospective sources, including literature and chart reviews, plus pretreatment data from clinical studies and company-supported EAPs. The comparable disease burden across the ISE-UPD and the ISE-pretreatment groups justified merging them to create a unified TK2d natural history dataset.

Provision of a timely and accurate diagnosis, which allows for early access to appropriate management strategies, is a common challenge faced by patients with a rare disease.^22^ The median diagnostic delay observed in this study was substantially longer for patients with age of symptom onset >12 years (delay of over 13 years) than for those with age of symptom onset ≤12 years (delay of just over 2 years), likely reflecting faster recognition in rapidly progressive early-onset disease, although some younger patients may die before receiving genetic confirmation of disease. The fact that *TK2* has only recently been added to nuclear gene panels for myopathies, and it is still not included in some other gene panels, adds to the challenge of timely diagnosis. Next-generation sequencing technologies have become readily available in recent years and may aid earlier recognition and diagnosis moving forward.

Our analysis of survival confirms the high mortality of TK2d. While the proportion of patients who died was greatest among those with earlier ages of symptom onset, the increased risk of early death was evident across all age-of-symptom-onset subgroups. Kaplan–Meier estimate for median time from symptom onset to death was 2.6 years in patients with age of symptom onset ≤12 years, and 24.0 years in patients with age of symptom onset >12 years, indicating substantial risk despite previous standards of care and specialist expertise.

A key novel contribution of this study is the quantification of developmental motor milestone achievement and loss for patients with TK2d, highlighting its substantial impact and relentless progression. Patients with age of symptom onset >12 years usually follow normal milestone attainment, although subtle symptoms of myopathy, such as slow running and weakness in the arms and legs, may be present in childhood.^1^ For patients with age of symptom onset ≤12 years, failure to achieve milestones may signal TK2d, or other diseases, and warrants further clinical investigation. It is worth noting that patients diagnosed below the age of 2 years may not yet have been expected to achieve all the developmental motor milestones assessed, complicating the interpretation of these data.

Loss of previously achieved motor milestones is a key impact of TK2d, especially for patients with age of symptom onset ≤12 years. In this group of patients, over 80% lost at least one motor milestone, with a median age of 2 years at first milestone loss; milestones were lost across the spectrum of motor development. Notably, the abilities to sit, stand or walk unassisted were each lost by roughly half of these patients. Even in patients with age of symptom onset >12 years, approximately one-third lost the ability to run, climb stairs unassisted or walk unassisted. Patients can have clinically significant muscle weakness despite retaining some of the motor functions examined here, and often report symptoms such as extreme fatigue or severe exercise intolerance.^23^ These data reflect that the disease course of TK2d is characterized by a lack of further achievement of higher developmental motor milestones and a loss of previously achieved milestones, without spontaneous recovery, confirming the unrelenting progressive disease nature.

Breathing difficulties, including shortness of breath on activity, sleep apnoea and respiratory failure, are reported to be the most impactful symptoms of TK2d on health-related quality of life and activities of daily living by patients, and on demands for caregivers as well as healthcare utilization.^24,25^ In our study, ventilatory support was used across all age-of-symptom-onset subgroups and the progressive increase in likelihood of using ventilatory support over time was apparent. Nevertheless, despite being present in all subgroups, the actual use of ventilatory support is likely underreported.

Although feeding tube support was observed across all subgroups, interpretation was limited by extensive missing data. Use of a feeding tube varies: some countries initiate support earlier than others, and some countries experience access issues. Data on the use of ventilatory and feeding tube support by patients with TK2d demonstrated increased risk over time and a high disease burden, which continues to accumulate after losing motor milestones. In patients with age of symptom onset ≤12 years with available data, ventilatory and feeding tube support were often initiated after the loss of key motor milestones.

Owing to the nature of our study design, some limitations were present. For some outcomes, missing values affecting over half of the population reduced the statistical power, reflecting the retrospective and difficult-to-collect nature of some data, consistent with other natural history studies of rare diseases.^26,27^ To avoid immortal-time bias, the ISE pretreatment group was excluded from survival analyses; however, treatment eligibility may introduce selection bias for functional outcomes. Further, the requirement for genetic confirmation of a TK2d diagnosis may impact estimated prevalence across age-of-symptom-onset subgroups. Pooling heterogeneous sources and study designs adds variability; we therefore focused on objective, discrete outcomes and did not retrospectively standardize motor or pulmonary function tests. Differences in median onset age between the ISE-UPD and the ISE pretreatment groups and older UPD data (from 2007) in the context of evolving diagnostics and standards of care may affect time-to-event comparisons. Despite these constraints, all analyses consistently demonstrate the high disease burden, relentlessly progressive nature, and increased risk of early mortality associated with TK2d.

Our study builds on previous reports of smaller patient groups^1,4,10,28-31^ and represents a substantial advance in the body of knowledge for TK2d. By capturing data for 257 patients with TK2d from around the world, this study represents a high proportion of the known global patient population, reducing the potential for bias that can result from case selection. Additionally, this study provides a critical benchmark for the evaluation of emerging therapies in TK2d. By quantifying survival, the progressive loss and rare regain of developmental motor milestones and the use of ventilatory and feeding support, these findings support the use of such outcomes as meaningful efficacy endpoints in future clinical trials. The stratification by age of symptom onset enables more precise study design and subgroup analysis, ensuring that therapeutic effects are appropriately contextualized. Furthermore, this dataset offers a robust comparator for single-arm studies. Ultimately, these results will help to ensure that future trials are designed to address the most clinically relevant outcomes in TK2d. Previous standards of care for TK2d had a limited impact on disease trajectory, highlighting the critical unmet treatment need that existed in TK2d. The first US FDA- and EMA-approved treatment for TK2d, doxecitine and doxribtimine, targets the underlying disease pathology. Data suggest that treatment with doxecitine and doxribtimine is well tolerated and can cause a positive change in TK2d disease trajectory and improved survival.^14,17^

In conclusion, our findings confirm the previously reported high mortality and progressive burden of TK2d, manifested as loss of motor milestones and use of ventilatory and feeding tube support (a plain language summary of these findings is provided in the supplementary materials to support understanding among patients, families and non-specialist audiences). Further, there is a continuum of clinically meaningful mortality and morbidity across differing ages of TK2d symptom onset. Patients with age of symptom onset ≤12 years appear to suffer from a more severe form of the disease than those with age of symptom onset >12 years. Our findings are likely to be of value to inform study designs for investigations in TK2d and contextualize the efficacy of new therapeutic options, including doxecitine and doxribtimine. By expanding the understanding of the disease course in untreated TK2d and highlighting unmet needs, we provide a strong foundation for further research into therapeutic strategies that could meaningfully improve patient outcomes in the future.

## Supplementary Material

fcag200_Supplementary_Data

## Data Availability

Data from non-interventional studies are outside of UCB’s data sharing policy and are unavailable for sharing. The data are not publicly available as they contain information that could compromise the privacy of participants.
