# Supplementary material for: Disease burden of untreated thymidine kinase 2 deficiency: insights from a large patient dataset
Source: Brain Commun. 2026 Jun 3;8(3):fcag200. doi: 10.1093/braincomms/fcag200 (PMC13273418; doi:10.1093/braincomms/fcag200)
Supplement: fcag200_Supplementary_Data [file fcag200_supplementary_data.docx]

# Supplementary Material

**Figures and Tables**

**Supplementary Figure 1 Kaplan–Meier plots of time to death from birth and from symptom onset in patients with TK2d from the MT-1621-107 untreated and updated-UPD groups.**


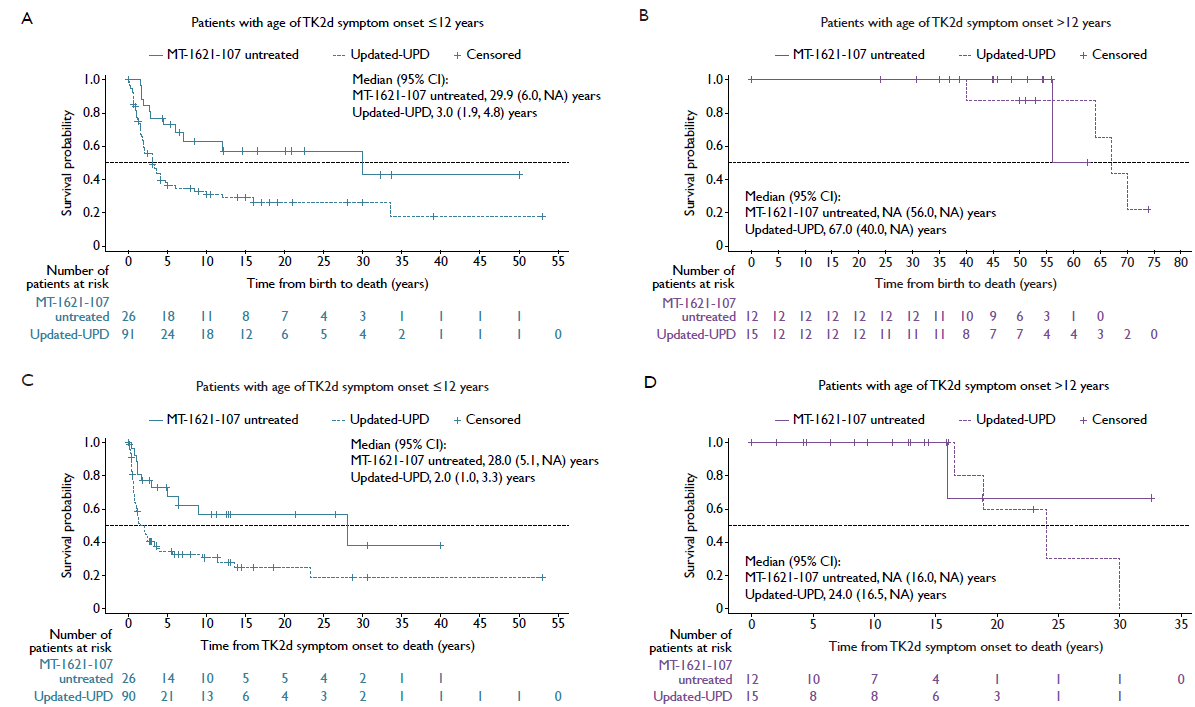


(**A**) Estimated survival time from birth to death in patients with age of symptom onset ≤12 years (MT-1621-107, *n* = 26; Updated-UPD, *n* = 91); (**B**) Estimated survival time from birth to death in patients with age of symptom onset >12 years (MT-1621-107, *n* = 12; Updated-UPD, *n* = 15); (**C**) Estimated survival time from symptom onset to death in patients with age of symptom onset ≤12 years (MT-1621-107, *n* = 26; Updated-UPD, *n* = 90); (**D**) Estimated survival time from symptom onset to death in patients with age of symptom onset >12 years (MT-1621-107, *n* = 12; Updated-UPD, *n* = 15)

CI = confidence interval; NA = not applicable; TK2d = thymidine kinase 2 deficiency; UPD = Untreated Patient Database.

**Supplementary Figure 2** **Kaplan–Meier plot of time from symptom onset to first loss of any motor milestone in patients with age of symptom onset ≤12 years.**


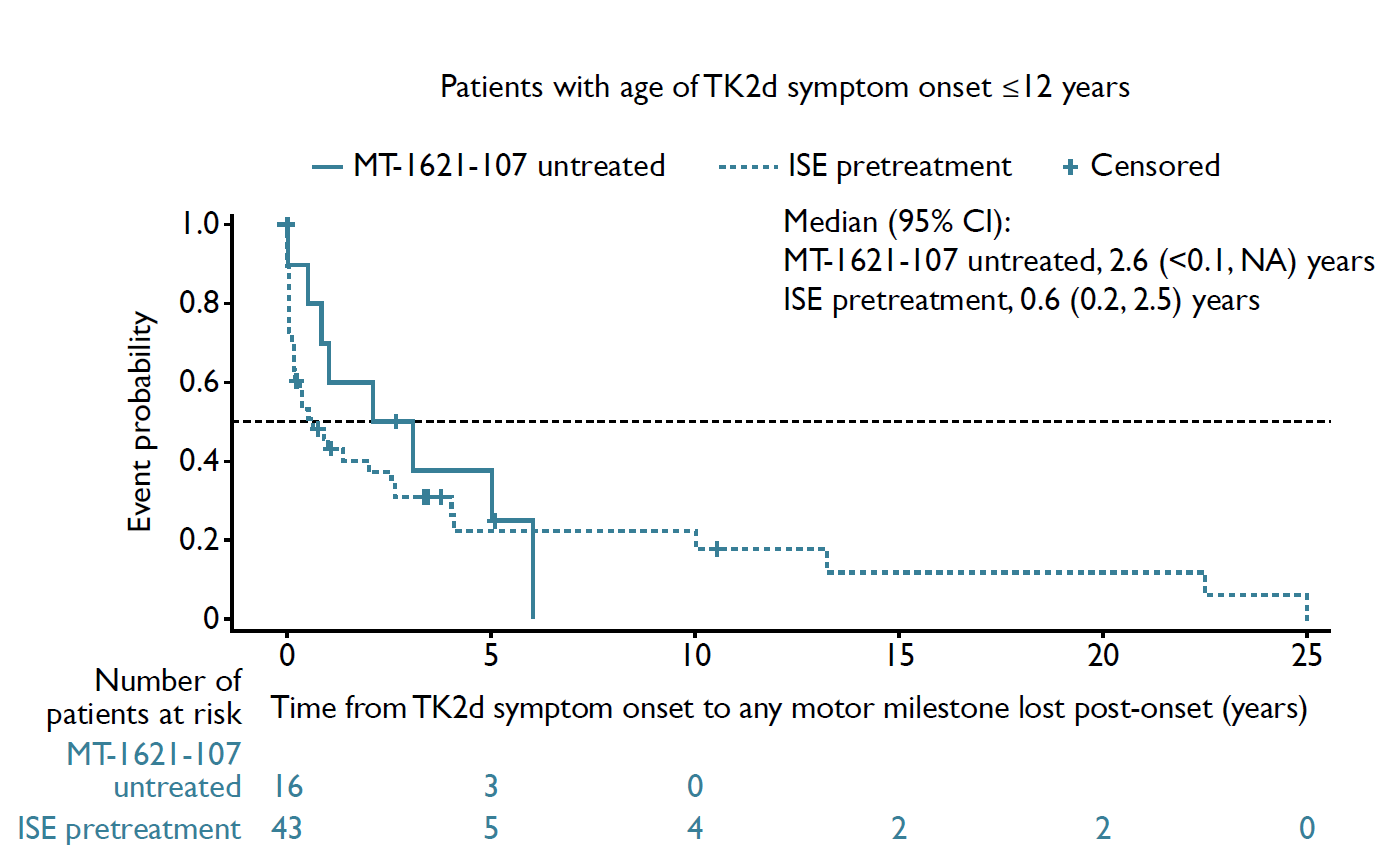


Estimated time from symptom onset to any motor milestone lost post-onset (MT-1621-107, *n* = 16; ISE pretreatment, *n* = 43). Within the ISE-UPD, motor milestone data were not available for any patients in the updated-UPD; consequently, only motor milestone data for the MT-1621-107 untreated group are shown.

A Kaplan–Meier plot of time from symptom onset to first loss of any motor milestone in patients with age of TK2d symptom onset >12 years could not be generated owing to the low number of patients at risk.

CI = confidence interval; ISE = Integrated Summary of Efficacy; NA = not applicable; TK2d = thymidine kinase 2 deficiency.

**Supplementary Figure 3** **Kaplan–Meier plots of time from symptom onset and from birth to first use of ventilatory support in different analysis populations.**


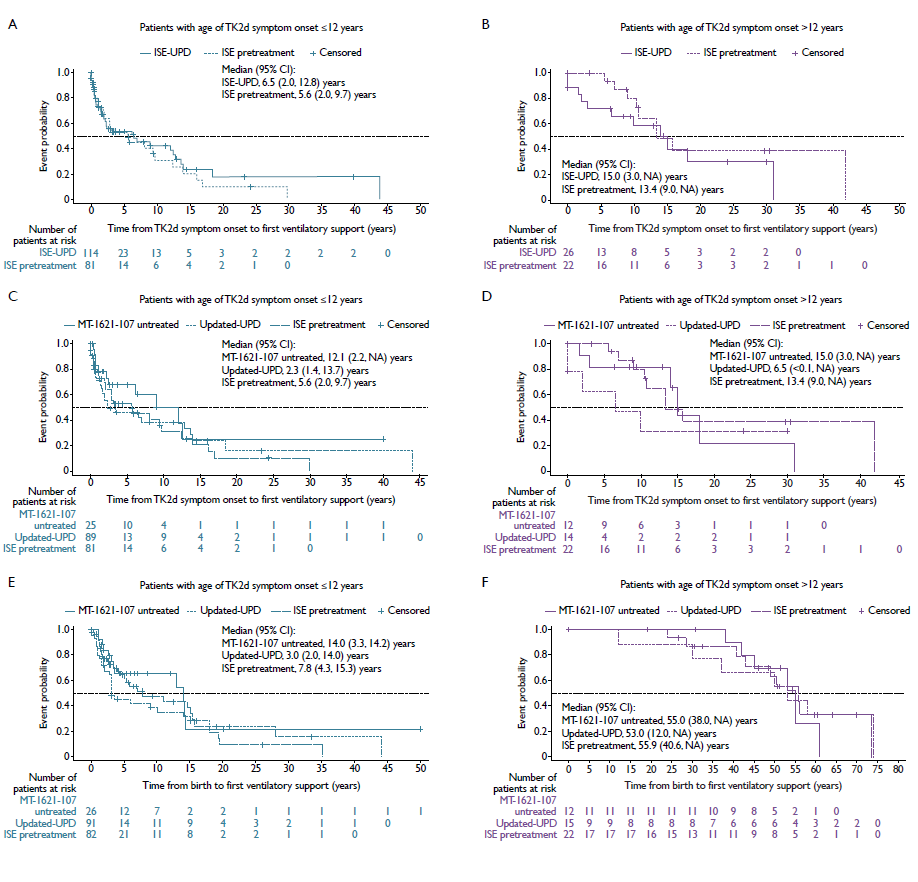


(**A, C**) Estimated time from symptom onset to first ventilatory support in patients with age of symptom onset ≤12 years (ISE-UPD, *n* = 114; ISE pretreatment, *n* = 81; MT-1621-107, *n* = 25; Updated-UPD, *n* = 89); (**B, D**) Estimated time from symptom onset to first ventilatory support in patients with age of symptom onset >12 years (ISE-UPD, *n* = 26; ISE pretreatment, *n* = 22; MT-1621-107, *n* = 12; Updated-UPD, *n* = 14); (**E**) Estimated time from birth to first ventilatory support in patients with age of symptom onset ≤12 years (MT-1621-107, *n* = 26; Updated-UPD, *n* = 91; ISE pretreatment, *n* = 82); (**F**) Estimated time from birth to first ventilatory support in patients with age of symptom onset >12 years (MT-1621-107, *n* = 12; Updated-UPD, *n* = 15; ISE pretreatment, *n* = 22)

The updated-UPD and MT-1621-107 untreated group were sub-analysis populations within the ISE-UPD.

CI = confidence interval; ISE = Integrated Summary of Efficacy; NA = not applicable; TK2d = thymidine kinase 2 deficiency; UPD, Untreated Patient Database.

**Supplementary Figure 4** **Kaplan–Meier plots of time from TK2d symptom onset to first use of feeding support.**


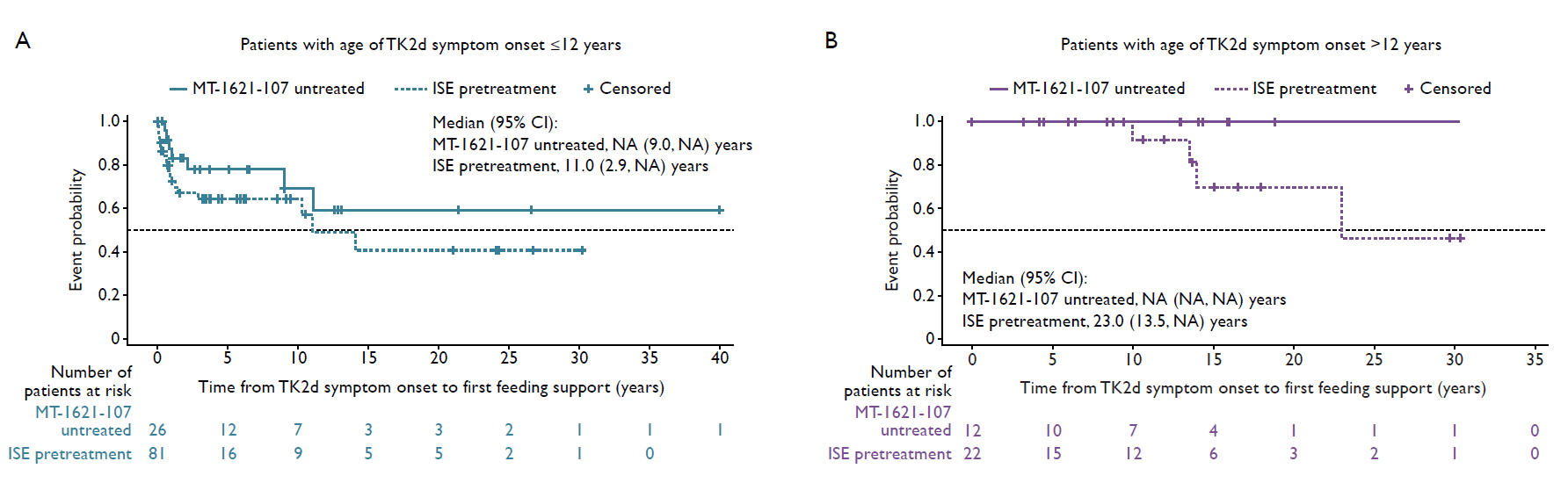


(**A**) Estimated time from symptom onset to first feeding support in patients with age of symptom onset ≤12 years (MT-1621-107, *n* = 26; ISE pretreatment, *n* = 81); (**B**) Estimated time from symptom onset to first feeding support in patients with age of symptom onset >12 years (MT-1621-107, *n* = 12; ISE pretreatment, *n* = 22).

Within the ISE-UPD, feeding support data were not available for any patients in the updated-UPD; consequently, only feeding support data for the MT-1621-107 untreated group are shown.

CI = confidence interval; ISE = Integrated Summary of Efficacy; NA = not applicable; TK2d = thymidine kinase 2 deficiency; UPD, Untreated Patient Database.

Supplementary Table 1 Literature search strategy

| **Search term** | **PubMed search term** |
| --- | --- |
| Thymidine kinase 2 deficiency | (“thymidine kinase 2”[Supplementary Concept] OR “thymidine kinase 2”[All Fields]) AND (“deficiency”[Subheading] OR “deficiency”[All Fields]) |
| TK2 | TK2[All Fields] |
| Thymidine kinase 2 | “thymidine kinase 2”[Supplementary Concept] OR “thymidine kinase 2”[All Fields] |
| TK2 mitochondrial DNA maintenance defects | TK2[All Fields] AND (“dna, mitochondrial”[MeSH Terms] OR (“dna”[All Fields] AND “mitochondrial”[All Fields]) OR “mitochondrial dna”[All Fields] OR (“mitochondrial”[All Fields] AND “dna”[All Fields])) AND (“maintenance”[MeSH Terms] OR “maintenance”[All Fields]) AND (“abnormalities”[Subheading] OR “abnormalities”[All Fields] OR “defects”[All Fields]) |

TK2 = thymidine kinase 2.

Supplementary Table 2 Data contributing to the characterization of the TK2d disease course

| **Data Source** | **Population** | **Number of unique patients^a^** | **Design** | **Data collection method** | **Data collection timepoints** | **Data contributing to course of disease evaluation** |
| --- | --- | --- | --- | --- | --- | --- |
| **Untreated patient group^b^** | | | | | | |
| Updated-UPD (Includes patients from NCT03701568) | Patients with TK2d identified via published comprehensive literature reviews, case series and case reports, and with patient-level data and genetic confirmation of a TK2d diagnosis, who were not treated with pyrimidine nucleos(t)ides | *N* = 113 | NA – All studies included irrespective of the study design | Literature review | NA – Limited to data available in publications | Demographic and disease characteristics, survival, ventilatory support status |
| MT-1621-107  (NCT05017818) | Patients with genetically confirmed TK2d, who were not treated with pyrimidine nucleos(t)ides | *N* = 40 | Phase 2, noninterventional, multicentre medical chart review program to collect vital status data and related information on treated and untreated patients with TK2d from studies outside those conducted by the Sponsor | Medical chart review | Longitudinal course of untreated patients | Demographic and disease characteristics, survival, motor milestones (acquisition, loss, regain, net gain), ventilatory support status, feeding support status |
| **Pretreated patient group** | | | | | | |
| MT-1621-101 (NCT03701568) | Patients with genetically confirmed TK2d treated with non-GMP dC and dT or dCMP and dTMP | *N* = 38 | Phase 2, multicentre, retrospective, noninterventional medical chart review | Medical chart review | Longitudinal, pretreatment course | Demographic and disease characteristics, survival, motor milestones (acquisition, loss, regain, net gain), ventilatory support status, feeding support status |
| TK0102 (NCT03845712) | Patients with genetically confirmed TK2d who previously participated in MT-1621-101 and patients treated with non-GMP dC and dT or dCMP and dTMP or doxecitine and doxribtimine (GMP-grade dC and dT) who did not participate in MT-1621-101 | *N* = 12 | Phase 2, prospective, open-label treatment program of the efficacy and safety of doxecitine and doxribtimine in patients with TK2d | Medical chart review for the patients carried over from MT-1621-101  Pretreatment data collected at screening visit for unique patients | Longitudinal, pretreatment course | Demographic and disease characteristics, survival, motor milestones (acquisition, loss, regain, net gain), ventilatory support status, feeding support status |
| MT-1621-107 (NCT05017818) | Patients with genetically confirmed TK2d treated with non-GMP dC and dT or dCMP and dTMP or doxecitine and doxribtimine (GMP-grade dC and dT) outside of a study conducted by the Sponsor | *N* = 17 | Phase 2, noninterventional, multicentre medical chart review program to collect vital status data and related information on treated and untreated patients with TK2d from studies outside those conducted by the sponsor | Medical chart review | Longitudinal pretreatment course | Demographic and disease characteristics, survival, motor milestones (acquisition, loss, regain, net gain), ventilatory support status, feeding support status |
| Company-supported Expanded Access Program | Patients with genetically confirmed diagnosis of TK2d who are at risk of major disability or death resulting from TK2d ad who are not eligible to participate in a clinical study conducted by the sponsor | *N* = 37 | Expanded access program | Pretreatment data collected at screening and follow-up | Pretreatment course | Demographic and disease characteristics, survival |

Note: The Comprehensive Disease Course Analysis Set was global in nature and included children, adolescents and adults from multiple countries of residence (including, but not limited to, USA, Spain, Israel, United Kingdom, Italy, Russia, Mexico and Turkey).

^a^The overall patient group analysis set includes those from the untreated patient group and those from the pretreatment patient group. Only unique patients were included in analyses, e.g. patients who were originally in the updated-UPD but were treated at a later point in time were analysed as part of the pretreatment group, utilizing all data up until the time treatment was initiated.

^b^Supplemental data collection for known untreated patients was performed to capture missing data, mainly on birth year/month and country of residence.

dC = deoxycytidine; dCMP = deoxycytidine monophosphate; dT = deoxythymidine; dTMP = deoxythymidine monophosphate; NA = not applicable; *TK2* = thymidine kinase 2 gene; TK2d = thymidine kinase 2 deficiency; UPD = Untreated Patient Database.

Supplementary Table 3 Baseline demographics and characteristics for patients stratified by age of TK2d symptom onset across all study analysis populations

| **Baseline demographics and characteristics** |  |  | **CDC** | | |
| --- | --- | --- | --- | --- | --- |
|  | **ISE-UPD** | | | **ISE pretreatment** | **CDC total** |
|  | **MT-1621-107 untreated** | **Updated-UPD** | **ISE-UPD total** |  |  |
| *Patients with age of symptom onset ≤12 years,^a^ N* | *26* | *91* | *117* | *82* | *199* |
| Sex, n (%)  Male  Female  Missing | 13 (50.0)  13 (50.0)  0 (0) | 49 (53.8)  40 (44.0)  2 (2.2) | 62 (53.0)  53 (45.3)  2 (1.7) | 46 (56.1)  36 (43.9)  0 (0) | 108 (54.3)  89 (44.7)  2 (1.0) |
| Race, n (%)  White  Other  Missing | 24 (92.3)  2 (7.7)  0 (0) | 0 (0)  0 (0)  91 (100) | 24 (20.5)  2 (1.7)  91 (77.8) | 67 (81.7)  11 (13.4)  4 (4.9) | 91 (45.7)  13 (6.5)  95 (47.7) |
| Ethnicity, n (%)  Hispanic or Latino  Not Hispanic or Latino  Missing | 12 (46.2)  14 (53.8)  0 (0) | 0 (0)  0 (0)  91 (100) | 12 (10.3)  14 (12.0)  91 (77.8) | 30 (36.6)  41 (50.0)  11 (13.4) | 42 (21.1)  55 (27.6)  102 (51.3) |
| Geographic region of residence,^b^ n (%)  Europe  Rest of world  Missing | 4 (15.4)  22 (84.6)  0 (0) | 16 (17.6)  26 (28.6)  49 (53.8) | 20 (17.1)  48 (41.0)  49 (41.9) | 27 (32.9)  55 (67.1)  0 (0) | 47 (23.6)  103 (51.8)  49 (24.6) |
| Age at TK2d symptom onset, years  Median (min, max)  Q1, Q3 | n = 26  1.6 (0.5, 10.0)  0.9, 3.9 | n = 91  1.0 (0.0, 11.0)  0.5, 2.0 | n = 117  1.2 (0.0, 11.0)  0.5, 2.0 | n = 82  1.5 (0.0, 11.7)  1.1, 2.4 | n = 199  1.4 (0.0, 11.7)  0.8, 2.3 |
| Age at genetic confirmation, years  Median (min, max)  Q1, Q3 | n = 26  6.7 (1.1, 56.4)  2.9, 15.5 | n = 33  4.4 (0.0, 43.6)  1.4, 13.0 | n = 59  5.2 (0.0, 56.4)  2.0, 14.4 | n = 77  3.2 (0.1, 35.3)  1.6, 8.3 | n = 136  4.1 (0.0, 56.4)  1.7, 10.3 |
| Time from TK2d symptom onset to genetic confirmation, months  Median (min, max)  Q1, Q3 | n = 26  65.8 (0.4, 556.4)  13.2, 129.1 | n = 33  30.1 (−5.9, 523.1)  6.1, 126.1 | n = 59  38.1 (−5.9, 556.4)  9.4, 129.1 | n = 77  12.3 (−59.9, 359.9)  4.3, 64.7 | n = 136  24.7 (−59.9, 556.4)  6.3, 90.1 |
|  |  |  |  |  |  |
| *Patients with age of TK2d symptom onset >12 years,^a^ N* | *12* | *15* | *27* | *22* | *49* |
| Sex, n (%)  Male  Female  Missing | 5 (41.7)  7 (58.3)  0 (0) | 4 (26.7)  9 (60.0)  2 (13.3) | 9 (33.3)  16 (59.3)  2 (7.4) | 9 (40.9)  13 (59.1)  0 (0) | 18 (36.7)  29 (59.2)  2 (4.1) |
| Race, n (%)  White  Other  Missing | 10 (83.3)  0 (0)  2 (16.7) | 0 (0)  0 (0)  15 (100) | 10 (37.0)  0 (0)  17 (63.0) | 20 (90.9)  2 (9.1)  0 (0) | 30 (61.2)  2 (4.1)  17 (34.7) |
| Ethnicity, n (%)  Hispanic or Latino  Not Hispanic or Latino  Missing | 1 (8.3)  10 (83.3)  1 (8.3) | 0 (0)  0 (0)  15 (100) | 1 (3.7)  10 (37.0)  16 (59.3) | 0 (0)  20 (90.9)  2 (9.1) | 1 (2.0)  30 (61.2)  18 (36.7) |
| Geographic region of residence,^b^ n (%)  Europe  Rest of world  Missing | 10 (83.3)  2 (16.7)  0 (0) | 2 (13.3)  2 (13.3)  11 (73.3) | 12 (44.4)  4 (14.8)  11 (40.7) | 16 (72.7)  6 (27.3)  0 (0) | 28 (57.1)  10 (20.4)  11 (22.4) |
| Age at TK2d symptom onset, years  Median (min, max)  Q1, Q3 | n = 12  40.0 (16.4, 50.0)  30.5, 42.7 | n = 15  37.0 (12.0, 72.0)  22.3, 50.0 | n = 27  40.0 (12.0, 72.0)  23.5, 45.0 | n = 22  27.1 (12.4, 60.3)  17.8, 40.0 | n = 49  31.0 (12.0, 72.0)  20.0, 40.0 |
| Age at genetic confirmation, years  Median (min, max)  Q1, Q3 | n = 12  47.3 (29.6, 59.9)  42.4, 53.7 | n = 4  35.7 (22.0, 75.5)  25.5, 58.9 | n = 16  44.9 (22.0, 75.5)  39.9, 53.7 | n = 22  48.1 (15.0, 73.6)  29.6, 57.8 | n = 38  46.3 (15.0, 75.5)  30.4, 56.8 |
| Time from TK2d symptom onset to genetic confirmation, months  Median (min, max)  Q1, Q3 | n = 12  127.9 (17.4, 358.3)  45.7, 197.8 | n = 4  213.1 (119.5, 328.6)  159.3, 277.9 | n = 16  152.8 (17.4, 358.3)  80.6, 216.2 | n = 22  172.5 (3.5, 524.0)  119.0, 311.1 | n = 38  159.1 (3.5, 524.0)  97.0, 271.4 |
|  |  |  |  |  |  |
| *Patients with age of TK2d symptom onset ≤2 years,^a^ N* | *17* | *73* | *90* | *56* | *146* |
| Sex, n (%)  Male  Female  Missing | 9 (52.9)  8 (47.1)  0 (0) | 37 (50.7)  34 (46.6)  2 (2.7) | 46 (51.1)  42 (46.7)  2 (2.2) | 31 (55.4)  25 (44.6)  0 (0) | 77 (52.7)  67 (45.9)  2 (1.4) |
| Race, n (%)  White  Other  Missing | 15 (88.2)  2 (11.8)  0 (0) | 0 (0)  0 (0)  73 (100) | 15 (16.7)  2 (2.2)  73 (81.1) | 43 (76.8)  9 (16.1)  4 (7.1) | 58 (39.7)  11 (7.5)  77 (52.7) |
| Ethnicity, n (%)  Hispanic or Latino  Not Hispanic or Latino  Missing | 7 (41.2)  10 (58.8)  0 (0) | 0 (0)  0 (0)  73 (100) | 7 (7.8)  10 (11.1)  73 (81.1) | 18 (32.1)  29 (51.8)  9 (16.1) | 25 (17.1)  39 (26.7)  82 (56.2) |
| Geographic region of residence,^b^ n (%)  Europe  Rest of world  Missing | 2 (11.8)  15 (88.2)  0 (0) | 13 (17.8)  19 (26.0)  41 (56.2) | 15 (16.7)  34 (37.8)  41 (45.6) | 18 (32.1)  38 (67.9)  0 (0) | 33 (22.6)  72 (49.3)  41 (28.1) |
| Age at TK2d symptom onset, years  Median (min, max)  Q1, Q3 | n = 17  1.2 (0.5, 2.0)  0.9, 1.5 | n = 73  0.8 (0.0, 2.0)  0.4, 1.3 | *n* = 90  0.9 (0.0, 2.0)  0.5, 1.5 | *n* = 56  1.2 (0.0, 2.0)  0.9, 1.5 | *n* = 146  1.1 (0.0, 2.0)  0.5, 1.5 |
| Age at genetic confirmation, years  Median (min, max)  Q1, Q3 | n = 17  5.3 (1.1, 23.9)  2.0, 7.7 | n = 23  2.4 (0.0, 43.6)  1.1, 6.7 | *n* = 40  3.1 (0.0, 43.6)  1.4, 7.2 | *n* = 52  2.3 (0.6, 28.8)  1.3, 5.4 | *n* = 92  2.4 (0.0, 43.6)  1.4, 6.7 |
| Time from TK2d symptom onset to genetic confirmation, months  Median (min, max)  Q1, Q3 | n = 17  52.3 (0.4, 266.4)  10.4, 70.0 | n = 23  21.5 (−5.9, 523.1)  3.5, 67.3 | *n* = 40  23.0 (−5.9, 523.1)  6.7, 69.4 | *n* = 52  10.4 (1.3, 327.3)  4.1, 57.3 | *n* = 92  17.5 (−5.9, 523.1)  5.2, 67.1 |
|  |  |  |  |  |  |
| *Patients with age of TK2d symptom onset >2 to ≤12* *years,^a^ N* | *9* | *18* | *27* | *26* | *53* |
| Sex, n (%)  Male  Female  Missing | 4 (44.4)  5 (55.6)  0 (0) | 12 (66.7)  6 (33.3)  0 (0) | 16 (59.3)  11 (40.7)  0 (0) | 15 (57.7)  11 (42.3)  0 (0) | 31 (58.5)  22 (41.5)  0 (0) |
| Race, n (%)  White  Other  Missing | 9 (100)  0 (0)  0 (0) | 0 (0)  0 (0)  18 (100) | 9 (33.3)  0 (0)  18 (66.7) | 24 (92.3)  2 (7.7)  0 (0) | 33 (62.3)  2 (3.8)  18 (34.0) |
| Ethnicity, n (%)  Hispanic or Latino  Not Hispanic or Latino  Missing | 5 (55.6)  4 (44.4)  0 (0) | 0 (0)  0 (0)  18 (100) | 5 (18.5)  4 (14.8)  18 (66.7) | 12 (46.2)  12 (46.2)  2 (7.7) | 17 (32.1)  16 (30.2)  20 (37.7) |
| Geographic region of residence,^b^ n (%)  Europe  Rest of world  Missing | 2 (22.2)  7 (77.8)  0 (0) | 3 (16.7)  7 (38.9)  8 (44.4) | 5 (18.5)  14 (51.9)  8 (29.6) | 9 (34.6)  17 (65.4)  0 (0) | 14 (26.4)  31 (58.5)  8 (15.1) |
| Age at TK2d symptom onset, years  Median (min, max)  Q1, Q3 | n = 9  4.0 (2.5, 10.0)  3.9, 7.0 | n = 18  3.8 (2.5, 11.0)  3.0, 5.0 | *n* = 27  4.0 (2.5, 11.0)  3.0, 7.0 | *n* = 26  2.8 (2.0, 11.7)  2.4, 5.0 | *n* = 53  3.6 (2.0, 11.7)  2.6, 5.3 |
| Age at genetic confirmation, years  Median (min, max)  Q1, Q3 | n = 9  13.8 (4.2, 56.4)  9.0, 17.7 | n = 10  10.0 (3.0, 32.3)  5.0, 20.0 | *n* = 19  13.0 (3.0, 56.4)  5.0, 20.0 | *n* = 25  5.8 (0.1, 35.3)  3.1, 12.6 | *n* = 44  9.0 (0.1, 56.4)  4.4, 15.2 |
| Time from TK2d symptom onset to genetic confirmation, months  Median (min, max)  Q1, Q3 | n = 9  107.6 (8.0, 556.4)  61.2, 129.1 | n = 10  78.1 (−5.9, 273.2)  30.1, 129.1 | *n* = 19  101.7 (−5.9, 556.4)  30.1, 129.1 | *n* = 25  33.9 (−59.9, 359.9)  7.8, 90.4 | *n* = 44  62.9 (−59.9, 556.4)  8.8, 121.5 |

^a^Age of TK2d symptom onset could not be determined for 9 patients; therefore, their data could not be included in this table.

^b^Owing to the ultra-rare nature of TK2d and the small number of patients, some details relating to race and country of residence were grouped for reporting purposes to minimize risk of patient identification.

ISE = Integrated Summary of Efficacy; max = maximum; min = minimum; Q1 = quartile 1; Q3 = quartile 3; TK2d = thymidine kinase 2 deficiency; UPD = Untreated Patient Database.

Supplementary Table 4 Summary of survival analysis for patients in the ISE-UPD group with age of symptom onset ≤2 years and >2 to ≤12 years

| **Summary of time to death** | **Age of TK2d symptom onset** | |
| --- | --- | --- |
|  | **≤2 years**  **(*n* = 90)** | **>2 to ≤12 years**  **(*n* = 27)** |
| **Patient status, n (%)**  Alive  Deceased  Missing data | 23 (25.6)  60 (66.7)  7 (7.8) | 18 (66.7)  6 (22.2)  3 (11.1) |
| **Age at death, years**  Mean (SD)  Median (min, max)  Q1, Q3 | *n* = 60  2.8 (4.2)  1.7 (0.0, 29.9)  1.0, 3.2 | *n* = 6  13.0 (11.1)  10.3 (3.0, 33.5)  4.8, 16.0 |
| **Kaplan–Meier estimates for time from birth to death**  Q1 (95% CI), years  Median (95% CI), years  Q3 (95% CI), years  Patients censored, n (%) | 1.3 (0.8, 1.6)  2.8 (1.8, 3.5)  10.0 (4.0, NA)  30 (33.3) | 33.5 (3.0, NA)  NA (16.0, NA)  NA (33.5, NA)  21 (77.8) |
| **Kaplan–Meier estimates for TK2d symptom onset to death**  Q1 (95% CI), years  Median (95% CI), years  Q3 (95% CI), years  Patients censored, n (%) | 0.6 (0.4, 0.8)  1.3 (1.0, 2.5)  6.4 (3.0, NA)  30 (33.7)^a^ | 13.5 (0.5, NA)  NA (13.5, NA)  NA (23.3, NA)  21 (77.8) |

^a^One patient was not at risk at point in time 0.

CI = confidence interval; ISE = Integrated Summary of Efficacy; max = maximum; min = minimum; NA = not applicable; Q1 = quartile 1; Q3 = quartile 3; SD = standard deviation; TK2d = thymidine kinase 2 deficiency; UPD = Untreated Patient Database.

Supplementary Table 5 Summary of motor milestones initially achieved by patients in the Comprehensive Disease Course group^a^ with age of symptom onset ≤2 years and >2 to ≤12 years, and those lost over the course of disease progression

| **Developmental motor milestone summary** | **Age of TK2d symptom onset** | |
| --- | --- | --- |
|  | ≤2 years (*n* = 146) | >2 to ≤12 years (*n* = 53) |
| **Patients with data for at least one response for milestones initially achieved, n (%)**  Missing data | 50 (34.2)  96^a^ (65.8) | 28 (52.8)  25^a^ (47.2) |
| **Patients with number of milestones initially achieved, n (%)**  ≥1 milestone  1 milestone  2 milestones  3 milestones  ≥4 milestones | 47 (94.0)  5 (10.0)  3 (6.0)  7 (14.0)  32 (64.0) | 28 (100)  0 (0)  0 (0)  0 (0)  28 (100) |
| **Patients with milestones initially achieved, n/N (%)**  Ability to hold head upright, unassisted  Ability to sit upright, unassisted  Ability to stand, assisted  Ability to stand, unassisted  Ability to walk, assisted  Ability to walk, unassisted  Ability to climb stairs, assisted  Ability to climb stairs, unassisted  Ability to run | 44/45 (97.8)  39/44 (88.6)  34/41 (82.9)  31/42 (73.8)  30/44 (68.2)  32/47 (68.1)  19/43 (44.2)  8/43 (18.6)  9/42 (21.4) | 28/28 (100)  28/28 (100)  28/28 (100)  28/28 (100)  26/27 (96.3)  28/28 (100)  26/26 (100)  19/26 (73.1)  20/26 (76.9) |
| **Patients with data for assessing milestone loss,^b^ n (%)**  Missing data | 47 (32.2)  99^a^ (67.8) | 28 (52.8)  25^a^ (47.2) |
| **Patients with milestones lost, n (%)**  ≥1 milestone  1 milestone  2 milestones  3 milestones  ≥4 milestones | 40 (85.1)  8 (17.0)  7 (14.9)  7 (14.9)  18 (38.3) | 21 (75.0)  4 (14.3)  6 (21.4)  1 (3.6)  10 (35.7) |
| **Age at which first milestone was lost, years**  Mean (SD)  Median (min, max)  Q1, Q3 | *n* = 34  2.8 (4.7)  1.3 (0.5, 24.2)  1.0, 2.0 | *n* = 19  7.0 (6.3)  4.4 (2.0, 27.3)  3.0, 10.0 |
| **Milestones lost, n (%)**  Ability to hold head upright, unassisted  Ability to sit upright, unassisted  Ability to stand, assisted  Ability to stand, unassisted  Ability to walk, assisted  Ability to walk, unassisted  Ability to climb stairs, assisted  Ability to climb stairs, unassisted  Ability to run | 28/44 (63.6)  21/39 (53.8)  20/34 (58.8)  18/31 (58.1)  17/30 (56.7)  20/32 (62.5)  13/19 (68.4)  7/8 (87.5)  7/9 (77.8) | 6/28 (21.4)  6/28 (21.4)  8/28 (28.6)  10/28 (35.7)  10/26 (38.5)  11/28 (39.3)  14/26 (53.8)  15/19 (78.9)  15/20 (75.0) |

^a^Data are presented for the Comprehensive Disease Course group; however, motor milestone data were not available for any patients in the updated-UPD (age of symptom onset ≤2 years, *n* = 73; age of symptom onset >2 to ≤12 years, *n* = 18) and for some patients in the ISE pretreatment group (age of symptom onset ≤2 years, *n* = 23; age of symptom onset >2 to ≤12 years, *n* = 7).

^b^Patients needed to have initially achieved at least one milestone to be assessed for milestone loss.

ISE = Integrated Summary of Efficacy; max = maximum; min = minimum; Q1= quartile 1; Q3 = quartile 3; SD = standard deviation; TK2d = thymidine kinase 2 deficiency; UPD = Untreated Patient Database.

Supplementary Table 6 Regain of lost motor milestones in patients in the Comprehensive Disease Course group^a^ by age of TK2d symptom onset

| **Regain of developmental motor milestones lost** | **Age of TK2d symptom onset** | | | |
| --- | --- | --- | --- | --- |
|  | **≤12 years**  **(*n* = 199)** | **>12 years**  **(*n* = 49)** | **≤2 years**  **(*n* = 146)** | **>2 to ≤12 years**  **(*n* = 53)** |
| **Patients with data for assessing milestone regain,^b^ n (%)**  Milestone not lost or missing data | 61 (30.7)  138^a^ (69.3) | 10 (20.4)  39^a^ (79.6) | 40 (27.4)  106^a^ (72.6) | 21 (39.6)  32^a^ (60.4) |
| **Number of milestones regained, n (%)**  ≥1  1  2  3  ≥4 | 3 (4.9)  3 (4.9)  0 (0)  0 (0)  0 (0) | 0 (0)  0 (0)  0 (0)  0 (0)  0 (0) | 2 (5.0)  2 (5.0)  0 (0)  0 (0)  0 (0) | 1 (4.8)  1 (4.8)  0 (0)  0 (0)  0 (0) |
| **Milestones regained, n (%)**  Ability to hold head upright, unassisted  Ability to sit upright, unassisted  Ability to stand, assisted  Ability to stand, unassisted  Ability to walk, assisted  Ability to walk, unassisted  Ability to climb stairs, assisted  Ability to climb stairs, unassisted  Ability to run | 0/34 (0)  0/27 (0)  1/28 (3.6)  0/28 (0)  0/27 (0)  1/31 (3.2)  0/27 (0)  0/22 (0)  1/22 (4.5) | 0  0  0  0  0  0/1 (0)  0  0/3 (0)  0/9 (0) | 0/28 (0)  0/21 (0)  1/20 (5.0)  0/18 (0)  0/17 (0)  1/20 (5.0)  0/13 (0)  0/7 (0)  0/7 (0) | 0/6 (0)  0/6 (0)  0/8 (0)  0/10 (0)  0/10 (0)  0/11 (0)  0/14 (0)  0/15 (0)  1/15 (6.7) |

^a^Data are presented for the Comprehensive Disease Course group; however, motor milestone data were not available for any patients in the updated-UPD (age of symptom onset ≤12 years, *n* = 91; age of symptom onset >12 years, *n* = 15; age of symptom onset ≤2 years, *n* = 73; age of symptom onset >2 to ≤12 years, *n* = 18) and for some patients in the MT-1621-107 untreated group (age of symptom onset ≤12 years, *n* = 0; age of symptom onset >12 years, *n* = 1; age of symptom onset ≤2 years, *n* = 0; age of symptom onset >2 to ≤12 years, *n* = 0) and the ISE pretreatment group (age of symptom onset ≤12 years, *n* = 30; age of symptom onset >12 years, *n* = 5; age of symptom onset ≤2 years, *n* = 23; age of symptom onset >2 to ≤12 years, *n* = 7).

^b^Patients needed to have lost at least one milestone to be assessed for milestone regain.

ISE = Integrated Summary of Efficacy; TK2d = thymidine kinase 2 deficiency; UPD = Untreated Patient Database.

Supplementary Table 7 Summary of ventilatory support and feeding support for patients in the Comprehensive Disease Course group^a^ with age of symptom onset ≤2 years and >2 to ≤12 years

| **Ventilatory and feeding tube support** | **Age of TK2d symptom onset** | |
| --- | --- | --- |
|  | **≤2 years**  **(*n* = 146)** | **>2 to ≤12 years**  **(*n* = 53)** |
| **Ventilatory support used at any time, n (%)**  Missing ventilatory support data | 58 (39.7)  63 (43.2) | 23 (43.4)  10 (18.9) |
| **Age at first ventilatory support, years**  Mean (SD)  Median (min, max)  Q1, Q3 | *n* = 53  4.3 (7.1)  1.7 (0.0, 44.0)  1.1, 3.1 | *n* = 20  12.6 (8.5)  12.0 (2.8, 35.2)  5.6, 16.7 |
| **Mode of first ventilatory support, n (%)**  Invasive (tracheostomy or no tracheostomy)  Non-invasive (e.g. BiPAP, CPAP)  Missing data | *n* = 58  12 (20.7)  17 (29.3)  29 (50.0) | *n* = 23  3 (13.0)  11 (47.8)  9 (39.1) |
| **Amount of ventilatory support used, hours/day**  Mean (SD)  Median (min, max)  Q1, Q3 | *n* = 26  17.5 (7.1)  23.0 (8.0, 24.0)  10.0, 24.0 | *n* = 10  13.4 (6.8)  10.0 (8.0, 24.0)  8.0, 22.0 |
| **Feeding tube (gastrostomy/nasogastric) support used at any time, n (%)**  Missing feeding tube support data | 23 (15.8)  96^a^ (65.8) | 5 (9.4)  25^a^ (47.2) |
| **Age at first feeding support, years**  Mean (SD)  Median (min, max)  Q1, Q3 | *n* = 22  2.8 (3.9)  1.5 (0.5, 16.0)  1.0, 2.5 | *n* = 5  12.4 (4.2)  13.1 (5.2, 16.3)  13.0, 14.5 |
| **Tube insertion reason for first occurrence, n (%)**  Supplemental oral intake  Dysphagia  Dysphagia, supplemental oral intake  Other  Missing data | *n* = 23  2 (8.7)  12 (52.2)  4 (17.4)  5 (21.7)  0 (0) | *n* = 5  2 (40.0)  1 (20.0)  2 (40.0)  0 (0)  0 (0) |
| **Long-term use of feeding tube,^b^ n (%)** | 16 (69.6) | 1 (20.0) |

^a^Data are presented for the Comprehensive Disease Course group; however, feeding support data were not available for any patients in the updated-UPD (age of symptom onset ≤2 years, *n* = 73; age of symptom onset >2 to ≤12 years, *n* = 18) and for some patients in the ISE pretreatment group (age of symptom onset ≤2 years, *n* = 23; age of symptom onset >2 to ≤12 years, *n* = 7).

^b^Long-term was defined as usage for over a month.

BiPAP = bilevel positive airway pressure; CPAP = continuous positive airway pressure; max = maximum; min = minimum; Q1 = quartile 1; Q3 = quartile 3; SD = standard deviation; TK2d = thymidine kinase 2 deficiency.

**Plain language summary**

Thymidine kinase 2 deficiency (TK2d) is a very rare disease that causes muscles to become weaker over time. People with TK2d often become unable to walk, eat or breathe without assistance, and are also at high risk of death at an early age. The disease is caused by changes in genes that are passed down from parents. TK2d reduces the ability of mitochondria (the ‘energy hubs’ of the cell) to produce energy. Information about how the signs and symptoms of this disease change over time is limited. Previous supportive care strategies did not change how the disease progressed. Doxecitine and doxribtimine, which works by targeting the root cause of TK2d, is the first approved treatment for patients with TK2d with symptoms starting at or before the age of 12 years in the USA and the European Union.

This study looked at signs and symptoms of TK2d in 257 untreated patients, representing the largest set of information about TK2d described so far. These data were collected from scientific reports and medical records of patients from around the world. The study describes the impact of the disease on life expectancy, motor abilities to perform physical movements (including sitting, standing and walking), and the need for medical equipment to help with breathing and feeding.

In this study, most patients with TK2d showed symptoms before the age of 12 years. The average life expectancy from the time their symptoms began was estimated to be 2.6 years for patients with symptoms before 12 years of age and 24 years for patients who first show symptoms after 12 years of age. More than half of the patients with symptoms that started before 12 years of age died (56%), while 22% of the patients with symptoms after 12 years of age died. It was more common for patients with symptoms before 12 years of age to lose some of the physical movement abilities that normally develop in childhood such as sitting, standing and walking (81.3% lost at least one physical movement ability), than for patients with first symptoms after 12 years of age (35.7% lost at least one physical movement ability). It was unusual for patients with TK2d to regain a physical movement ability once they had lost it. Patients in both groups in the study, those with symptoms before age of 12 years and those with symptoms starting after the age of 12 years, had used supportive equipment to help them with breathing and feeding.

This study highlights the serious effects that TK2d has on life expectancy and daily life, especially for those who develop symptoms at an early age.
